# Phytoconstituents, *In Vitro* Anti-Infective Activity of *Buddleja indica* Lam., and *In Silico* Evaluation of its SARS-CoV-2 Inhibitory Potential

**DOI:** 10.3389/fphar.2021.619373

**Published:** 2021-04-12

**Authors:** Fadia S. Youssef, Ahmed E. Altyar, Abdelsattar M. Omar, Mohamed L. Ashour

**Affiliations:** ^1^ Department of Pharmacognosy, Faculty of Pharmacy, Ain Shams University, Cairo, Egypt; ^2^ Department of Pharmacy Practice, Faculty of Pharmacy, King Abdulaziz University, Jeddah, Saudi Arabia; ^3^ Department of Pharmaceutical Chemistry, Faculty of Pharmacy, King Abdulaziz University, Jeddah, Saudi Arabia; ^4^ Department of Pharmaceutical Chemistry, Faculty of Pharmacy, Al-Azhar University, Cairo, Egypt; ^5^ Department of Pharmaceutical Sciences, Pharmacy Program, Batterjee Medical College, Jeddah, Saudi Arabia

**Keywords:** antimicrobial, antiviral, *Buddleia indica*, COVID-19, molecular modeling, phytoconstituents, Scrophulariaceae

## Abstract

Phytochemical investigation of *Buddleja indica* Lam. leaves methanol extract (BIT) resulted in the isolation of six known compounds for the first time from the plant, namely, *p*-hydroxybenzoic acid 1), caffeic acid 2), quercetin 3-O-*β-*D glucoside-7-O-α-L-rhamnoside 3), kaempferol 3-O-*β-*D glucoside-7-O-α-L-rhamnoside 4), quercetin 7-O-*β-*D glucoside 5) and kaempferol 6). BIT extract showed potent antibacterial activity with MIC values ranging between 0.48 and 1.95 μg/ml with *Bacillus subtilis* was the most susceptible to the BIT effect. It showed a notable antimycobacterial and anti-*Helicobacter pylori* activity with MIC values of 100 and 80 μg/ml, respectively. Vesicular stomatitis virus (VSV) was more sensitive to the antiviral activity of BIT comparable to herpes simplex virus type 1 (HSV-1), showing 48.38 and 41.85% inhibition of the viral replication at a dose of 50 μg/ml for VSV and HSV-1, respectively. *In silico* molecular docking of the isolated compounds revealed that caffeic acid 2) showed the highest fitting within the active sites of DNA-gyrase, topoisomerase IV, and SARS-CoV-2 M^Pro^. Quercetin 7-O-*β-*D glucoside 5) revealed the best fitting in dihydrofolate reductase active site with ∆ G value equals −36.53 Kcal/mol. Kaempferol 6) exhibited the highest fitting towards *β*-lactamase, SARS-CoV-2PL^pro^, and SARS-CoV-2 3CL^pro^ active sites. Thus, *B. indica* Lam. can be considered as a future source of cheap, substantially safe, and credible antibacterial, antifungal, and antiviral candidate of natural origin that could effectively participate in solving the problem of COVID-19 pandemic. These findings provide a scientific consolidation for the ethnomedicinal uses of *Buddleja indica* Lam. as a topical antiseptic.

## Introduction

In spite of the great progress in the therapeutic strategies for the alleviation of many human health disorders, infectious diseases due to bacteria, fungi, and viruses still constitute a major challenge to public health (Youssef et al., 2014; Ayoub et al., 2015). Recently, in late December 2019, a novel coronavirus strain impacted people in Wuhan, China, and they suffered from pneumonia and this virus was named severe acute respiratory syndrome–related coronavirus (SARS-CoV-2), resulting in a lethal respiratory disorder. People experiencing severe COVID-19 suffered from cytokine storm syndrome with concomitant secondary hemophagocytic, hyperinflammation, lymphohistiocytosis, hyperferritinemia, cytopenias, and acute respiratory distress syndrome. Hence, combating this pandemic and other infectious agents is of high necessity that demands the rapid discovery of effective drug entities (Mehta et al., 2020; Munnink et al., 2020). Plant kingdom constitutes an everlasting source of bioactive compounds that turn out to be relatively safe and widely acceptable by a large category of patients compared to synthetic drugs (Ashour et al., 2018; Thabet et al., 2018a).

**GRAPHICAL ABSTRACT:**
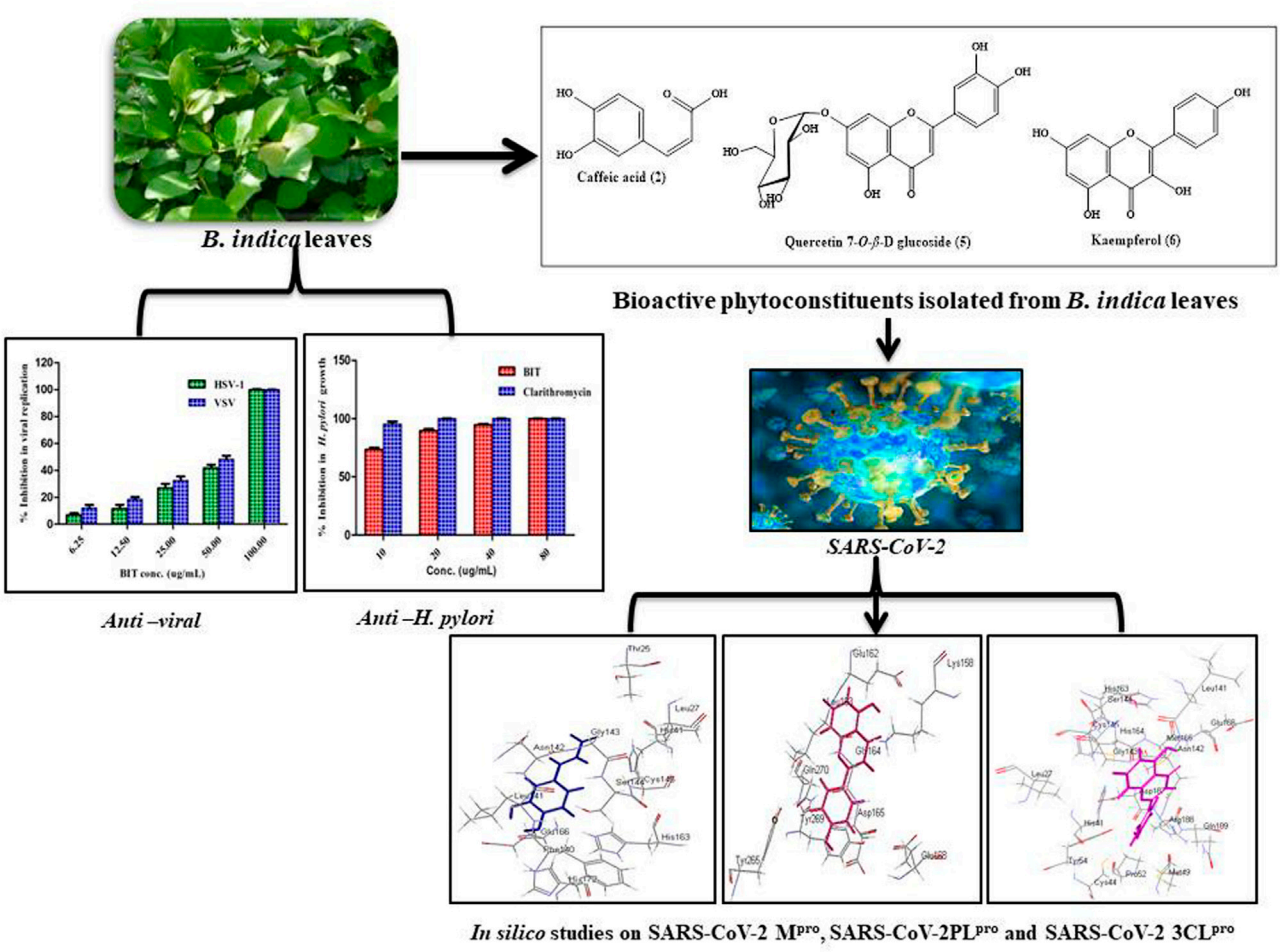


*Buddleja* (Buddleia) has recently been included in the Scrophulariaceae family. It comprises about 100 species growing natively in Africa, South and North America, and Asia; meanwhile, a considerable number of the species were cultivated all over the globe, especially in central Europe and New Zealand. The *Buddleja* genus is known for possessing several biological activities represented mainly by antiviral, antimicrobial, antipyretic, antioxidant, immunosuppressive, hepatoprotective, antihypertensive, analgesic, and antipyretic activities. This is undoubtedly owing to its richness with various secondary metabolites exemplified mainly by phenylpropanoids, flavonoids, and iridoid glucosides (Youssef et al., 2018; Youssef et al., 2019).

*Buddleja indica* Lam. is an ornamental evergreen shrub of African, mainly Madagascan, origin. In spite of the popularity of the *Buddleja* genus in many medicinal uses, nothing was found in the literature concerning the biological activity or the phytoconstituents of *B. indica* Lam. except for its antioxidant and hepatoprotective potential (Youssef et al., 2019). It is noteworthy to mention that many members of the *Buddleja* genus were traditionally used as topical antiseptics without any scientific consolidation.

This study aimed to identify the secondary metabolites of *B. indica* Lam. leaves methanol extract using different techniques. The antimicrobial activity against a panel of bacteria and fungi, the antimycobacterial, anti-*Helicobacter pylori*, and antiviral potential will be assessed using *in vitro* studies for the first time. Furthermore, the prospect of its utilization to alleviate the COVID-19 pandemic, which has recently attacked the world resulting in many deaths, will be evaluated using *in silico* studies. This will be performed using Discovery Studio software, aiming to explore its probable mode of action and exact behavior with the binding site of major *Coronavirus* proteins, if any. This will be done in hope of finding new natural resources that can be safely incorporated into pharmaceutical dosage forms aiming to solve this crisis.

## Materials and Methods

### Plant Material

*B. indica* Lam. was purchased from El-Orman Botanical Garden, Giza, Egypt, in 2019. Identification and authentication of the plant were kindly performed by Mrs. Theresa Labib, Consultant of Plant Taxonomy at the Ministry of Agriculture, Giza, Egypt. The voucher specimen of the plant was given a voucher number of PHG-P-BI-163 and kept at Pharmacognosy Department, Faculty of Pharmacy, Ain Shams University, Egypt.

### General Experimental Procedures

NMR spectral measurements were recorded on a Varian AS 500 MHz spectrometer. Polyamide 6 S (Fluka Analytical, Germany) and silica gel (60–120 mesh, Merck) were used for column chromatography (CC). Monitoring of CC fractions was done on precoated aluminum sheets [silica 60 F254, 0.25 mm (Merck, Darmstadt, Germany)], and detection was achieved using UV light at two different λ, 254 and 366 nm, that were concomitantly sprayed with 1% vanillin-sulfuric acid reagent and heated for 5–10 min at 105°C. HPLC (Knauer, Germany) supplied with semipreparative column Kromasil 100-5 C18 in addition to UV detector (K-2501) was used for further purification of the semipure fractions. All the solvents used are of high analytical grades, whereas those for HPLC analysis are of HPLC grade (Sigma-Aldrich (St. Louis, MO, United States).

### Preparation of *Buddleja indica* Lam. Leaf Extract

The air-dried leaves of *B. indica* Lam. (500 g) were coarsely ground and then macerated in 5 L of distilled methanol with subsequent filtration and this process was repeated three times till exhaustion. The collected filtrate was subsequently evaporated under reduced pressure at a temperature not exceeding 45°C and then lyophilized to give 120 g of dried total methanol extract (BIT).

### Isolation of Secondary Metabolites

About 105 g of the lyophilized extract was solubilized in 70% distilled methanol followed by fractionation using *n*-hexane, dichloromethane, and ethyl acetate successively to give 21.93, 8.32, and 27 g residue, respectively, with about 49.2 g remaining hydroalcoholic fraction. A 25 g of the ethyl acetate fraction was solubilized in the least amount of water and chromatographed over a polyamide S column chromatography and eluted with H_2_O/MeOH of decreasing polarity till reaching 100% MeOH. Similar fractions were pooled together after being monitored using TLC on silica gel 254 sheets that resulted in ten major fractions. Fraction III eluted with 30% methanol was further chromatographed on silica gel column using CH_2_Cl_2_: CH_3_OH of increasing polarity to give eight fractions. Fraction III-5 was subjected to preparative TLC using CH_2_Cl_2_: CH_3_OH (8:2) to yield compound 1) (2 mg) and compound 2) (3.5 mg). Fraction IV eluted with 40% methanol was further chromatographed on a silica gel column. Similar fractions were pooled together after being monitored by TLC on silica gel 254 sheets to give sixty fractions. Fraction IV-44 was subjected to preparative HPLC using gradient elution using acetonitrile: water solvent system with a flow rate of 4 ml/min. This process resulted in the isolation of compound 3) (2.5 mg) and compound 4) (1 mg). However, fraction V, eluted with 50% methanol, was further chromatographed on silica gel column and similar fractions were pooled together after being monitored by TLC on silica gel 254 sheets to give 18 fractions. Fraction V-4 was subjected to preparative TLC using CH_2_Cl_2_: CH_3_OH (9:1) to yield compound 5) (2 mg). Regarding fraction VIII, it was eluted with 80% methanol and was further chromatographed on silica gel column using CH_2_Cl_2_: CH_3_OH of increasing polarity to give ten fractions. Fraction VIII-1 was subjected to preparative TLC using CH_2_Cl_2_: CH_3_OH (9:1) to yield compound 6) (1.5 mg). A scheme representing the isolation of compounds (1–6) from *B. indica* Lam. total methanol leaves extract is shown in (Supplementary Figure S1) in the supplementary data.

### Spectroscopic Data of Compounds (1–6)

The spectral data of all the previously isolated compounds (1–6) included 1D and 2D NMR. The compounds were defined as *p*-hydroxybenzoic acid 1) (Ariga et al., 2015), caffeic acid 2) (Jeong et al., 2011), quercetin 3-O-*β*-D-glucoside-7-O-α-L-rhamnoside 3) (Gaind et al., 1981), kaempferol 3-O-*β*-D glucoside-7-O-α-L-rhamnoside 4) (Gaind et al., 1981), quercetin 7-O-*β*-D glucoside 5) (Gansukh et al., 2016), and kaempferol 6) (Hadizadeh et al., 2003). NMR spectral data were supplied in the supplementary data in Supplementary Figures S2–S19.

## Biological Assessments

### Assessment of the Antimicrobial Activity

#### Microbial Strains

BIT was investigated vs. a panel of microorganisms comprising standard Gram-positive bacteria, *Staphylococcus aureus* (ATCC 29213) and *Bacillus subtilis* (ATCC 6051); Gram-negative bacteria, *Pseudomonas aeruginosa* (ATCC 12525) and *Escherichia coli* (ATCC 25922); fungi, *Aspergillus fumigatus* (ATCC 1022), *Geotrichum candidum* (ATCC 12784), *Candida albicans* (ATCC 90028), and *Syncephalastrum racemosum* (ATCC 14831), at Al-Azhar University, Nasr City, Cairo, Egypt.

#### Determination of the Mean Inhibition Zones

Mean inhibition zones for the antibacterial potential were measured employing the previously described method by Damyanova et al. (Damyanova et al., 2000). Ampicillin and streptomycin were used as antibacterial standards at a concentration of 30 μg/ml. Meanwhile, the mean inhibition zones for the antifungal activity were determined using the method previously adopted by Rathore et al. (Rathore et al., 2000), in which clotrimazole and itraconazole at a concentration of 30 μg/ml were used as the positive controls.

#### Determination of the Minimum Inhibitory Concentration

MIC values of BIT vs. all the examined bacterial and fungal strains were determined using the agar plate method. Nutrient agar was used for bacterial strains, whereas Sabouraud dextrose agar was employed for fungi. The respective agar media were heated in the autoclave for 25 min at 121°C and allowed to cool to 45°C. Twofold serial dilutions of the tested sample were added to the medium immediately before being poured into the Petri dishes. DMSO was used as the negative control. The culture of each organism in the nutrient broth (beef extract 5 g/L and peptone 10 g/L, pH = 7.0) for bacteria and Sabouraud dextrose broth for fungi was diluted with sterile distilled water to 10^5^–10^6^ CFU/mL. A loop of each suspension was inoculated in the appropriate medium with the sample or the control added. After inoculation, the plates were incubated at 37°C for 24 h for bacteria and at 30°C for three to four days for fungi. The MIC was considered to be the lowest concentration that completely inhibited the visible growth of a microorganism compared with the control (Rahman et al., 2001). Each test was performed in triplicate and both streptomycin and clotrimazole were used as positive controls.

### Assessment of the Antimycobacterial Activity

The antimycobacterial potential of BIT was determined by the microplate Alamar blue assay (MABA). The used *M. tuberculosis* strain (RCMB 010126) was obtained from the culture collection of the Regional Center for Mycology and Biotechnology (RCMB), Al-Azhar University (Cairo, Egypt). This was performed as previously described and isoniazid was used as a standard drug (Gamal El-Din et al., 2018). Percent inhibition was defined as 1—(mean of test well/mean of B wells) × 100, whereas MIC was defined as the lowest concentration of drug that prevented this change in color.

### Assessment of the Anti-*Helicobacter pylori* Activity

The anti-*Helicobacter pylori* effectiveness of BIT was determined using *Helicobacter pylori* ATCC 43504 following the NCCLS guidelines (1998) and as previously described (Gamal El-Din et al., 2018). Clarithromycin was used as a positive control. Inhibition (%) was computed as follows: [(Initial control absorbance-final absorbance)/(Initial control absorbance)] × 100.

### Assessment of the Antiviral Activity

#### Cell Cultures

Vero cells (CCL-81) (Vero African green monkey kidney cells) were maintained in DMEM complete media with a high glucose level of 0.45% (L-glutamine supplemented with 10% heat-inactivated fetal bovine serum (FBS), 100 U/mL penicillin, and 100 U/ml streptomycin). Cells were cultured in 10 cm cell culture dishes (Cellstar) at 37°C in a humidified atmosphere of 5% CO_2_. The cells were maintained as “monolayer culture” by serial subculturing. All experiments were performed with cells in the logarithmic growth phase under strict aseptic conditions in a biosafety level 2 lab using a biological safety cabinet level 2 (Ashour et al., 2014).

#### Cytotoxicity and Cell Proliferation MTT Assay

Cytotoxicity was evaluated by applying the MTT cell viability assay (Van de Loosdrecht et al., 1991; Thabet et al., 2018b). Briefly, this assay depends on the conversion of MTT (3-(4,5-dimethylthiazol-2-yl)-2,5-diphenyltetrazolium bromide) by the viable cells from the yellow color to purple formazan compound. Vero cells (CCL-81) about 2 × 10^4^ cell/well in the exponential phase were seeded in a 96-well plate and they were cultivated for 24 h and then incubated with various concentrations of the serially diluted tested sample (stock solution 1 mg/ml) at 37°C for 24 h followed by their incubation with 0.5 mg/ml MTT for 4 h. The formed formazan compound was solubilized in 200 µL DMSO and the absorbance was measured at 570 nm. The cell viability rate (%) of three independent experiments was calculated by the following formula (Youssef et al., 2016):

### The Plaque Reduction Assay

The antiviral activity was determined by a plaque reduction assay (Abou-Karam & Shier, 1990). Briefly, a confluent layer of Vero cells (CCL-81) was obtained by culturing the cells for 24 h in 0.5% CO_2_ at 37°C. The cells were inoculated separately with herpes simplex virus type 1 (HSV-1) (ATCC VR 1493) or vesicular stomatitis virus (VSV) (ATCC VR 1283) (1 × 10^−1^–10^−7^/ml) and incubated at 37°C for 1 h. The infected cell cultures (2 × 10^3^ PFU) were washed and overlaid with DMEM containing five concentrations of BIT (6.25, 12.5, 25, 50, and 100 μg/ml) for 1 h at room temperature. Each concentration was performed in three replicates and cultures were overlaid with nutrient agarose (DMEM 2x/1.8% agarose [v/v]) containing 25 mM MgCl_2_. After 72 h incubation, cells were fixed with 10% formaldehyde in phosphate buffer, pH = 7.3, for 1 h, and stained with 0.5% crystal violet in 20% ethanol. The plaques were counted and the percentage of viral inhibition was calculated as [1−(V_*d*_/V_*c*_)] × 100, where V_*d*_ and V_*c*_ refer to the number of plaques in the presence and absence of the tested sample, respectively. Acyclovir was employed as a positive control. The viral strains HSV-1 and VSV are available at VACSERA, Giza, Egypt. Biosafety level II was strictly followed; all equipment, vials, viral cultures, stocks, and potentially infectious materials were properly decontaminated via autoclaving prior to disposal. In addition, protective clothing was worn during preparation to prevent contamination and to guard against harm (World Health Organization, 2004).

### *In Silico* Molecular Docking Studies

Molecular modeling studies were performed for the isolated phytoconstituents within the active centers incorporated in the occurrence of bacterial infection and antibiotic resistance development such as DNA-gyrase (PDB ID 4Z2D; 3.38 A°); topoisomerase IV (PDB ID 4Z3O; 3.44 A°); dihydrofolate reductase (PDB ID 4KM2; 1.4 A°); *β*-lactamase (PDB ID 3NBL; 2.0 A°). Additionally, the main target proteins required to prohibit COVID-19 replication, that is the main protease SARS-CoV-2 M^Pro^ (PDB ID: 6LZE; 1.50 A°), SARS-CoV-2 papain-like protease (PL^pro^) (PDB ID: 4OW0; 2.10 A°), and 3-chymotrypsin-like protease SARS-CoV-2 3CL^pro^ (PDB ID: 6M2N; 2.20 A°), were tested using Discovery Studio 4.5 (Accelrys Inc., San Diego, CA, USA) applying C-Docker protocol using both pH-based and rule-based methods that were chosen during the preparation of the ligand as an option in the software prior to performing the docking experiments as previously described (Ashour et al., 2018; Janibekov et al., 2018; Thabet et al., 2018). It is noteworthy to highlight that in the pH-based method, docking takes place under conditions that mimic the interaction in the physiological medium, whereas in the rule-based method, docking takes place regarding the functional groups only. The free binding energies (∆G) were calculated in Kcal/mol using the following equation:

$$\Delta {\text{G}}_{\text{binding}}={\text{E}}_{\text{complex}}-\left({\text{E}}_{\text{protein}}+{\text{E}}_{\text{ligand}}\right).$$

Here, ΔG_binding_ is the ligand–protein interaction binding energy, E_complex_ is the potential energy for the complex of protein bound with the ligand, E_protein_ is the potential energy of protein alone, and E_ligand_ is the potential energy for the ligand alone.

Moreover, the validation of molecular docking was performed for all the carried docking experiments via comparing the alignment of the best docking poses for the lead compound that varies according to the targeted enzyme with the lead conformer cocrystallized with the respective enzyme. RMSD (Root-Mean-Square Deviation) value is used to confirm the validity of the docking experiment and indicates the ability to predict the binding mode of novel ligands.

### Statistical Analysis

All the measurements were done in triplicate in three independent times. Data were expressed as the mean ± SD. Graphs were constructed by GraphPad Prism^®^ 5.1 (GraphPad Software, Inc., CA, United States). The *p* value < 0.05 was considered a significance difference between comparison groups.

## Results and Discussion

### Phytochemical Characterization

**FIGURE 1:**
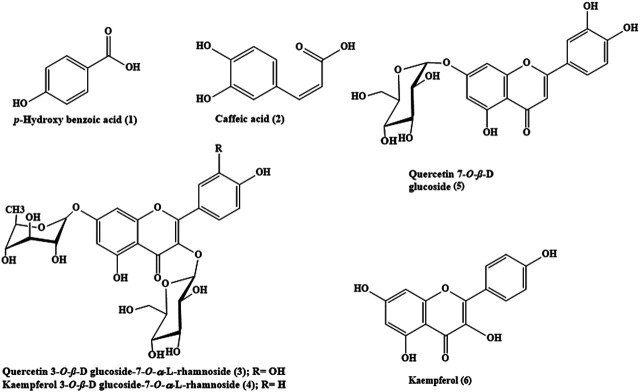
The chemical structures of phenolic compounds isolated from *B. indica* leaves.

Phytochemical investigation of *B. indica* Lam. leaves methanol extract resulted in the isolation and structural elucidation of six known polyphenolic compounds, which were the first to be isolated from the plant. They belong mainly to phenolic acids and flavonoids, which were fully elucidated and identified via comparing their 1D and 2D NMR data with what was previously reported in the literature. They were defined as *p*-hydroxybenzoic acid 1) (Ariga et al., 2015), caffeic acid 2) (Jeong et al., 2011), quercetin 3-O-*β*-D-glucoside-7-O-α-l-rhamnoside 3) (Gaind et al., 1981), kaempferol 3-O-*β*-D glucoside-7-O-α-l-rhamnoside 4) (Gaind et al., 1981), quercetin 7-O-*β*-D glucoside 5) (Gansukh et al., 2016), and kaempferol 6) (Hadizadeh et al., 2003). A scheme showing the compounds isolated from *B. indica* Lam. leaves is illustrated in Figure 1. It is noteworthy to mention that *p*-hydroxybenzoic acid, buddlenoid B, 2′-O-benzoyl aucubin, buddlejoside A, kaempferol-7-O-*α*-L-rhamnopyranoside, 6-acetylaucubin, gmelinoside F and gmelinoside H, isorhamnetin 7-O-*α*-L-rhamnopyranoside, catalpol 6-O-[4-methoxy-E-cinnamoyl-(3)-α-l-rhamnopyranoside, and acacetin-7-galactoside were previously determined by the authors from the plant extract using LC-ESI-MS (Youssef et al., 2019).

### The Antimicrobial, Antimycobacterial, and Anti-*Helicobacter pylori* Activity

The antibacterial activity of BIT was evaluated *in vitro* using the agar well diffusion method against different standard Gram-positive and Gram-negative bacteria via measuring the mean inhibition zones and determining the MICs. The values for the mean inhibition zones were illustrated in Table 1. BIT extract showed potent antibacterial activity against the tested Gram-positive and Gram-negative bacterial strains with MIC values ranging between 0.48 and 1.95 μg/ml. *Bacillus subtilis* was the most susceptible to the BIT effect, followed by *Staphylococcus aureus* and *Escherichia coli* displaying MICs of 0.48 µg/ml for the former and 0.97 μg/ml for the latter two (Table 2).

**TABLE 1:** Mean inhibition zones of BIT against different pathogens determined by the agar diffusion method.

	Microorganisms	Diameter of inhibition zone (mm)				
		BIT	Ampicillin	Streptomycin	Itraconazole	Clotrimazole
		(50 mg/ml)	(30 µg/ml)	(30 µg/ml)	(30 µg/ml)	(30 µg/ml)
Gram (+)						
	*Staphylococcus aureus* (ATCC 29213)	24.3 ± 0.02	30.1 ± 0.06	28.1 ± 0.07	NT	NT
	*Bacillus subtilis* (ATCC 6051)	25.7 ± 0.20	31.6 ± 0.05	29.7 ± 0.06	NT	NT
Gram (-)						
	*Pseudomonas aeruginosa* (ATCC 12525)	21.7 ± 0.04	28.3 ± 0.08	25.2 ± 0.09	NT	NT
	*Escherichia coli* (ATCC 25922)	24.9 ± 0.07	33.1 ± 0.09	29.7 ± 0.07	NT	NT
Fungi						
	*Aspergillus fumigatus* (ATCC 1022)	24.3 ± 0.04	NT	NT	27.4 ± 0.05	26.3 ± 0.08
	*Geotrichum candidum* (ATCC 12784)	21.5 ± 0.09	NT	NT	24.2 ± 0.09	23.2 ± 0.03
	*Candida albicans* (ATCC 90028)	19.4 ± 0.01	NT	NT	25.2 ± 0.07	20.8 ± 0.02
	*Syncephalastrum racemosum* (ATCC 14831)	17.5 ± 0.07	NT	NT	23.9 ± 0.04	21.4 ± 0.05

Data are measured in triplicate (n = 3) and presented as means ± SD. Well diameter: 6.0 mm (100 µL was tested). NT: not tested.

**TABLE 2:** Minimum inhibitory concentrations (MICs) of BIT against different pathogens determined by the agar plate method.

	Microorganisms	Minimum inhibitory concentration (MIC) (µg/ml)			
		BIT	Ampicillin	Streptomycin	Clotrimazole
Gram (+)					
	*Staphylococcus aureus* (ATCC 29213)	0.97	0.16	0.24	NT
	*Bacillus subtilis* (ATCC 6051)	0.48	0.08	0.12	NT
Gram (-)					
	*Pseudomonas aeruginosa* (ATCC 12525)	1.95	0.24	0.48	NT
	*Escherichia coli* (ATCC 25922)	0.97	0.06	0.12	NT
Fungi					
	*Aspergillus fumigatus* (ATCC 1022)	0.97	NT	NT	0.12
	*Geotrichum candidum* (ATCC 12784)	1.95	NT	NT	0.48
	*Candida albicans* (ATCC 90028)	3.9	NT	NT	0.97
	*Syncephalastrum racemosum* (ATCC 14831)	7.8	NT	NT	0.48

NT: Not tested.

Moreover, BIT extract was screened *in vitro* for its antifungal activity against various fungi on Sabourad dextrose agar plates. Then, the diameter of the inhibition zone (in mm) (Table 1) and the minimum inhibitory concentration (MIC) values were calculated. The extract showed a powerful inhibition against nearly all of the tested organisms, as evidenced by its MIC values approaching that of the clotrimazole, the standard antifungal agent. It is worthy of mentioning that BIT is highly effective against *Aspergillus fumigatus* followed by *Geotrichum candidum* and *Candida albicans* with MICs of 0.97, 1.95, and 3.9 μg/ml, respectively. Additionally, *Syncephalastrum racemosum* was moderately sensitive to the antimicrobial effect of BIT with a MIC value of 7.8 μg/ml (Table 2).

**FIGURE 2:**
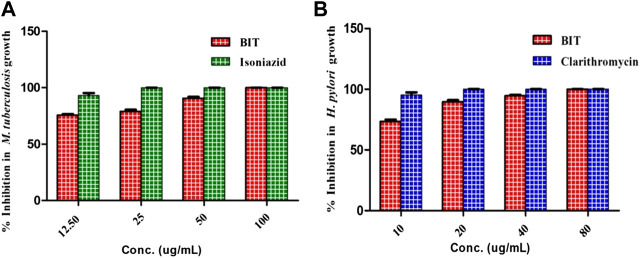
Effect of BIT and isoniazid different concentrations on the *M. tuberculosis* **(A)** and effect of different BIT concentrations and clarithromycin on *H. pylori* growth **(B)**.

Additionally, BIT extract showed substantial antimycobacterial activity against *M. tuberculosis,* exhibiting a MIC value of 100 μg/ml. It is noteworthy to mention that BIT extract and isoniazid showed 75.8 and 93.24% inhibition to *M. tuberculosis* growth at 12.5 μg/ml, respectively (Figure 2A). On the other hand, BIT extract also proved a notable activity against *Helicobacter pylori* growth with a MIC value of 80 μg/ml with respect to the positive control, clarithromycin, that showed a MIC value of 20 μg/ml. Furthermore, the extract showed 73.4% inhibition to *Helicobacter pylori* growth at 10 μg/ml compared to 95.37% inhibition shown by the positive control at the same concentration (Figure 2A).

Various extracts and isolated compounds from many *Buddleja* species were previously reported to possess a prominent antimicrobial activity. The methanol extracts of the leaves and stems of *B. saligna* produced effective inhibition to various Gram-positive and some Gram-negative microbial strains (Adedapo et al., 2009). Additionally, *B. saligna* hexane fraction showed a considerable bactericidal potency against *Mycobacterium tuberculosis* using bioautography and this could be explained in virtue of its DNA polymerase inhibitory activity (Bamuamba et al., 2008). In addition, *B. cordata* stem bark extract exhibited a notable antimycobacterial activity (Acevedo et al., 2000). Moreover, *B. globosa* and *B. cordata* leaves were reputed as potent antibacterial agents vs. *Staphylococcus aureus* and *Escherichia coli* due to the presence of various polyphenolic compounds as verbascoside that displayed MIC at a value of 1 μM (Guillermo Avila et al., 1999). However, the lipophilic, chloroform, extract of *B. globosa* stem bark displayed a potent antifungal activity with MIC of 125 μg/ml against *Epidermophyton floccosum, Trichophyton interdigitale*, and *Trichophyton rubrum* owing to the existence of terpenoid compounds, namely, buddlejone and buddledins A and B (Mensah et al., 2000). Furthermore, *B. perfoliata* Kunth that was popular in folk medicine for the alleviation of digestive tract disorder showed *in vitro* anti-*H. pylori* activity with MIC values for the aqueous and methanol extracts of its aerial parts of 500 and 62.5 μg/ml, respectively (Castillo-Juárez et al., 2009). In addition, *p*-hydroxybenzoic acid and caffeic acid were reported to possess a prominent antifungal and antibacterial activity vs. a wide array of bacteria and fungi (Manuja et al., 2013).

### The Antiviral Activity

**FIGURE 3:**
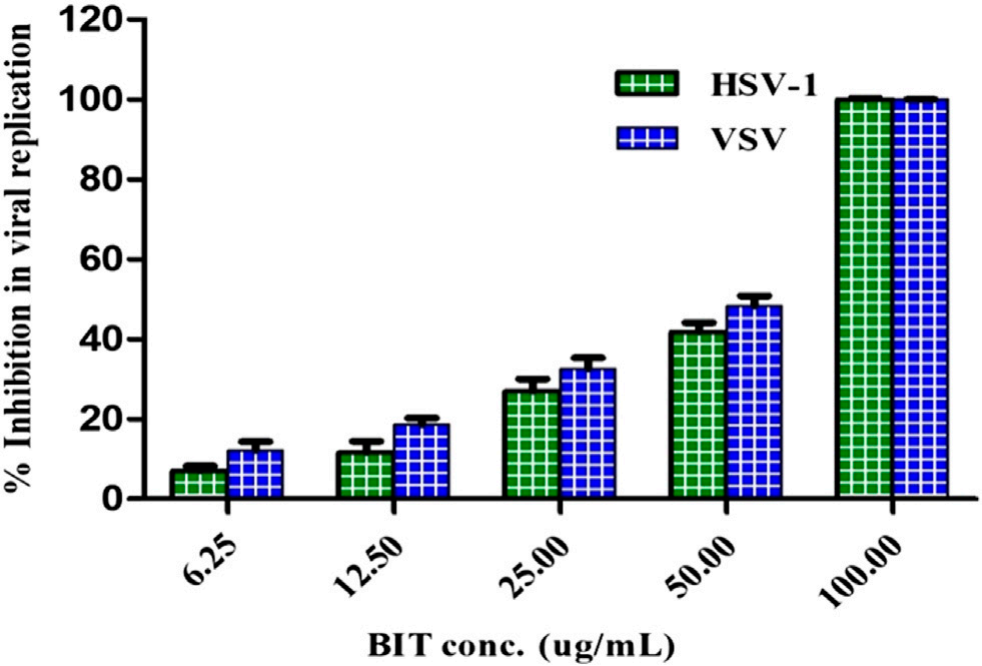
Effect of BIT different concentrations on HSV-1 and VSV growth using direct plaque reduction assay.

The antiviral activity was assessed using the plaque reduction assay on Vero cells (CCL-81). The BIT extract lacks measurable cytotoxicity on Vero cells at the selected doses with IC_50_ of 251.47 μg/mL as shown by the MTT assay. However, BIT showed notable antiviral activity in a dose-dependent manner against VSV and HSV-1, as demonstrated in Figure 3. Meanwhile, VSV was more sensitive to the antiviral activity of the BIT comparable to HSV-1, showing 48.38 and 41.85% inhibition of the viral replication at a dose of 50 μg/ml for VSV and HSV-1, respectively, with IC_50_ equaling 52.2 and 58.6 μg/ml for VSV and HSV-1, respectively, whereas acyclovir, the standard antiviral, demonstrated IC _50_ values of 2.21 and 1.49 μg/ml for VSV and HSV-1, respectively (Supplementary Table S1). It is noteworthy to mention that few reports were found considering the antiviral activity of various *Buddleja* species in which *B. cordobensis* essential oil showed modest activity against dengue virus type 2, herpes simplex virus type 1, and Junin virus (Duschatzky et al., 2005). However, compounds isolated from BIT showed antiviral activity. Caffeic acid isolated from BIT revealed potent anti-HBV, anti-HSV, and/or ADV activities in addition to the prohibition of human coronavirus NL63 (Chiang et al., 2002; Wang et al., 2009; Langland et al., 2018; Weng et al., 2019) caused by inhibition of viral DNA replication, antigen production, and reduction of virus level in the serum (Wang et al., 2009). Furthermore, quercetin 7-O-*β*-D-glucoside exhibited potent inhibitory potential vs. influenza A and B viruses via reducing ROS autophagy production and prohibiting viral RNA polymerase by occupying m7GTP on PB2 protein of the virus (Gansukh et al., 2016). In addition, kaempferol and its derivatives, particularly with rhamnose glycoside, strongly prohibited the 3a channel protein of SARS coronavirus and thus act as good candidates for 3a channel proteins of coronaviruses (Schwarz et al., 2014).

### *In Silico* Molecular Docking Studies

*In silico* molecular docking of the isolated compounds was performed on crucial proteins incorporated into the growth and replication of microbes and SARS-CoV-2 in the hope of finding promising candidates to fight infection and to solve the COVID-19 pandemic. In this study, four proteins were selected as they are critical for the survival, replication, and development of bacterial resistance, namely, DNA-gyrase (PDB ID 4Z2D; 3.38 A°); topoisomerase IV (PDB ID 4Z3O; 3.44 A°); dihydrofolate reductase (PDB ID 4KM2; 1.4 A°); *β*-lactamase (PDB ID 3NBL; 2.0 A°). Additionally, three crucial proteins for SARS-CoV-2, which represent ideal targets to prohibit viral replication, namely, main protease SARS-CoV-2 M^Pro^ (PDB ID: 6LZE; 1.50 A°), SARS-CoV-2 papain-like protease SARS-CoV-2PL^pro^) (PDB ID: 4OW0; 2.10 A°), and 3-chymotrypsin-like protease SARS-CoV-2 3CL^pro^ (PDB ID: 6M2N; 2.20 A°), were also examined using Discovery Studio 4.5 applying C-Docker protocol employing both pH-based and rule-based experiments in an effort to search for entities that could act as leads that may help in solving this crisis.

Results of the validation experiments revealed a good alignment between the best-docked poses of the lead compound, with the lead conformer cocrystallized with the respective enzymes showing RMSD values of 1.53, 1.05, 1.70, 2.00, 1.71, 2.10, and 2.30 A° for DNA-gyrase, topoisomerase IV, dihydrofolate reductase, *β*-lactamase, SARS-CoV-2 M^Pro^, SARS-CoV-2PL^pro^, and SARS-CoV-2 3CL^pro^, respectively, confirming the validity of the experiment. Figures showing the alignment between the best-docked poses of the lead compound with the lead conformer cocrystallized with the respective enzymes were displayed in Supplementary Figure S20.

**FIGURE 4:**
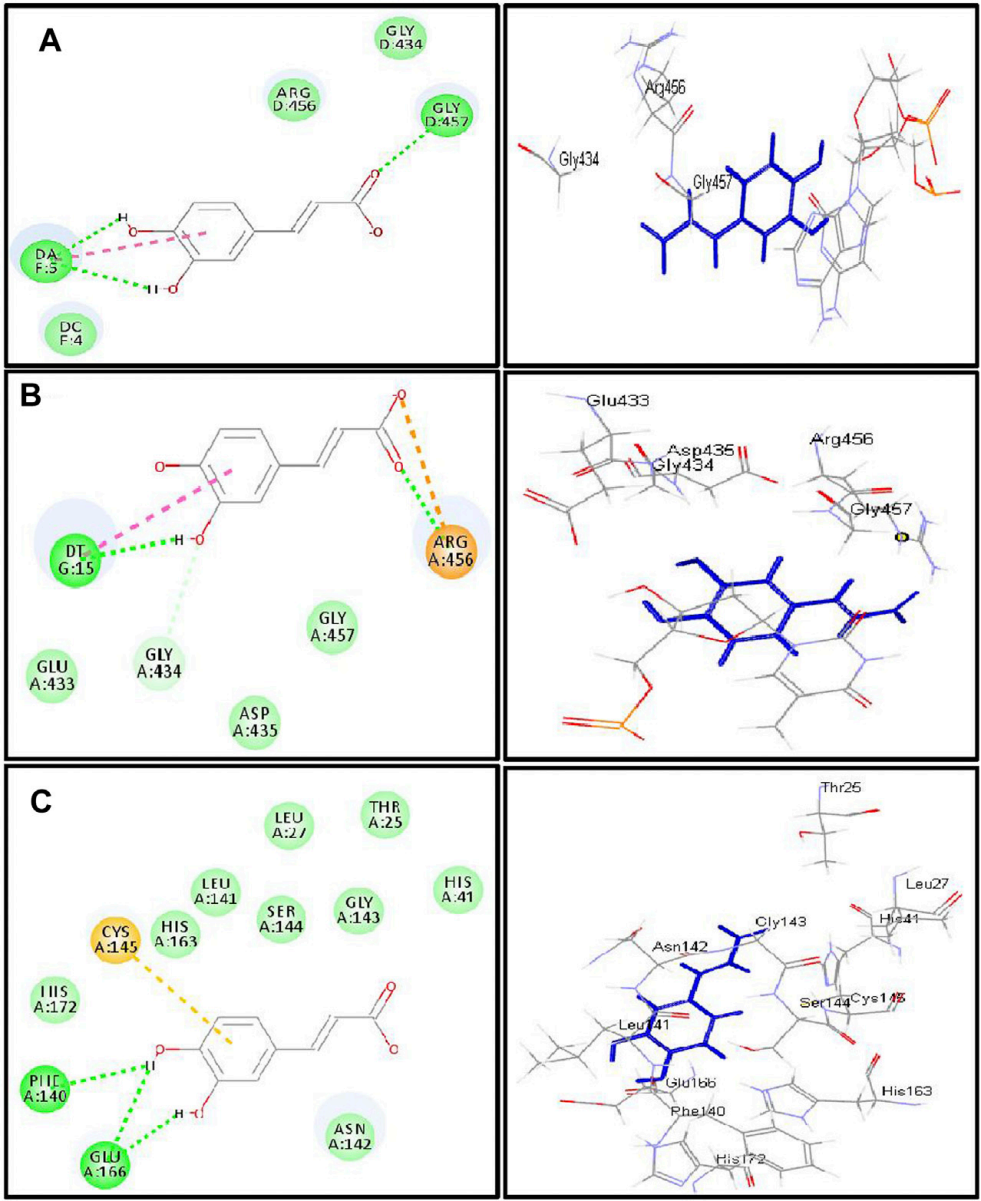
2D and 3D binding modes of caffeic acid in the active centers of **(A)** DNA-gyrase, **(B)** topoisomerase IV, and **(C)** SARS-CoV-2 M^Pro^; dotted green lines indicate H-bonds; dotted light green lines indicate C-H-bonds; dotted purple lines indicate π-bonds; dotted orange bonds indicate salt bridge formation.

**FIGURE 5:**
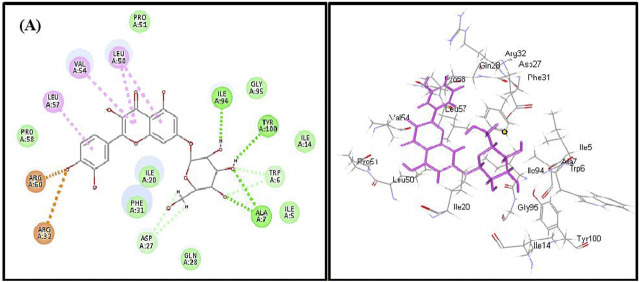
2D and 3D binding modes of quercetin 7-O-*β-D*-glucoside in dihydrofolate reductase active sites; dotted green lines indicate H-bonds; dotted light green lines indicate C-H-bonds; dotted purple lines indicate π-bonds; dotted orange bonds indicate salt bridge formation.

**FIGURE 6:**
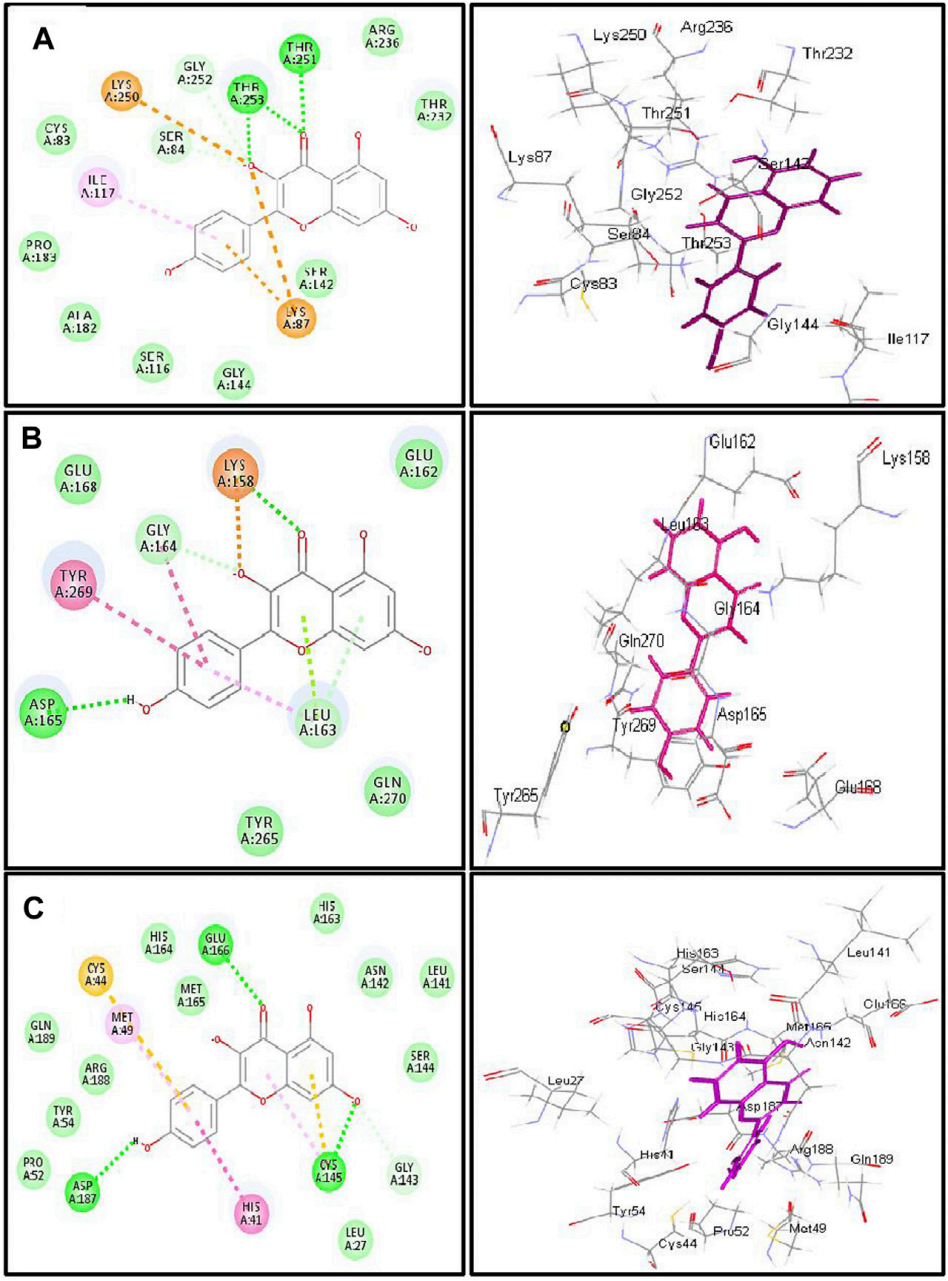
2D and 3D binding modes of kaempferol in the active centers of (A) β-lactamase (B) SARS-CoV-2PLpro and (C) SARS-CoV-2 3CLpro; dotted green lines indicate H-bonds; dotted light green lines indicate C-H-bonds; dotted purple lines indicate π-bonds; dotted orange bonds indicate salt bridge formation.

Employing pH-based docking that mimics the interaction in the physiological medium, results illustrated in Table 3 and Table 4 revealed that caffeic acid 2) showed the highest fitting within the active sites of DNA-gyrase, topoisomerase IV, and SARS-CoV-2 M^Pro^ displaying ∆G of -20.45, -25.65, and -28.13 Kcal/mol, respectively, showing superior activity to levofloxacin, moxifloxacin, and SARS-CoV-2 M^Pro^ ligand (FHR/PRD_002347) with free binding energies equal to -9.9, -10.22, and -4.60 kcal/mol, respectively. Meanwhile, quercetin 7-O-*β*-D-glucoside 5) revealed the best fitting in dihydrofolate reductase active site with ∆G value equal to −36.53 Kcal/mol exceeding that of trimethoprim that showed ∆ G of -28.90 kcal/mol. Furthermore, kaempferol 6) exhibited the highest fitting towards *β*-lactamase, SARS-CoV-2PL^pro^, and SARS-CoV-2 3CL^pro^ active sites with free binding energies equal to −46.92, −28.61, and −32.77 Kcal/mol, respectively, approaching in this aspect cefuroxime, main SARS-CoV-2 PL^pro^ ligand S88, and main SARS-CoV-2 3CL^pr^ ligand 3WL that exerted free binding energies equal to −61.76, −22.53, and −34.66 Kcal/mol, respectively. 2D and 3D binding modes of compounds showing the highest fitting score within SARS-CoV-2 M^Pro^, SARS-CoV-2PL^pro^, and SARS-CoV-2 3CL^pro^ are illustrated in Figures 4–6.

**TABLE 3:** Free binding energies (ΔG) in Kcal/mol of compounds isolated from *B. indica* Lam. leaves in the active sites of enzymes involved in the occurrence of bacterial infections and resistance using *in silico* studies.

Compounds	DNA−gyrase		Topoisomerase IV		Dihydrofolate reductase		*β*−lactamase	
	pH	Rule	pH	Rule	pH	Rule	pH	Rule
*p*−Hydroxy benzoic acid **(1)**	−13.34	−13.79	−18.37	−18.40	−15.83	−15.67	−35.98	−35.00
Caffeic acid **(2)**	−20.45	−20.45	−25.65	−25.65	−35.61	−35.61	−46.74	−46.74
Quercetin 3−O− *β*−D−glucoside−7−O− α−L−rhamnoside **(3)**	12.97	12.63	13.36	16.17	2.35	3.68	−16.33	−0.96
Kaempferol 3−O−*β−*D−glucoside−7−O− α−L−rhamnoside **(4)**	15.28	15.28	16.74	16.74	0.79	7.94	−1.78	−1.78
Quercetin 7−O− *β*−D glucoside **(5)**	−6.16	−13.24	−15.68	−11.43	−36.53	−18.94	−37.09	−24.16
Kaempferol **(6)**	−17.92	−21.44	−23.61	−20.94	−35.66	−27.85	−46.92	−30.08
Levofloxacin	−9.90	−9.90	ND	ND	ND	ND	ND	ND
Moxifloxacin	ND	ND	−10.22	−10.22	ND	ND	ND	ND
Trimethoprim	ND	ND	ND	ND	−28.90	−28.90	ND	ND
Cefuroxime	ND	ND	ND	ND	ND	ND	−61.76	−61.76

FD: fail to dock; ND: not done.

**TABLE 4:** Free binding energies (ΔG) in Kcal/mol of compounds isolated from *B. indica* Lam. leaves in the active sites of specific proteins that serve as the main targets for SARS-CoV-2 eradication employing *in silico* studies.

Compounds	SARS-CoV-2 M^Pro^		SARS-CoV-2PL^pro^		SARS-CoV-2 3CL^pro^	
	pH	Rule	pH	Rule	pH	Rule
*p*-Hydroxy benzoic acid **(1)**	−21.95	−21.00	−21.07	−20.99	−20.78	−17.10
Caffeic acid **(2)**	−28.13	−28.13	−26.79	−26.79	−28.53	−28.53
Quercetin 3-O-*β-*D-glucoside-7-O-α-l-rhamnoside **(3)**	−3.75	−1.70	13.91	20.96	−2.18686	8.05
Kaempferol 3-O-*β-*D-glucoside-7-O-α-l-rhamnoside **(4)**	7.71	8.61	34.88	34.88	11.13	12.29
Quercetin 7-O-*β-*D glucoside **(5)**	−19.76	−19.52	−11.40	−11.83	−22.86	−19.62
Kaempferol **(6)**	−27.84	−25.84	−28.61	−26.27	−32.77	−31.76
SARS-CoV-2 M^Pro^ ligand (FHR/PRD_002347)	−4.60	−4.60	ND	ND	ND	ND
SARS-CoV-2 PL^pro^ ligand S88	ND	ND	−22.53	−22.53	ND	ND
SARS-CoV-2 3CL^pr^ ligand 3WL	ND	ND	ND	ND	−34.66	−34.66

ND: not done.

The firm binding of caffeic acid at the active site of DNA-gyrase could be explained by virtue of the formation of three H-bonds, one π-π bond, and Van der Waals interactions with the amino acid residues existing at the active site. Similarly, the formation of two H-bonds, one π-π bond, C-H bond, and many Van der Waals interactions with amino acid existing at the active site of topoisomerase IV explained the tight binding of caffeic acid to its active center. Regarding the SARS-CoV-2 coronavirus, caffeic acid revealed a strong binding to main protease (SARS-CoV-2 M^Pro^) that is interpreted by the formation of three conventional H-bonds with Phe 140, Glu 166, and π-sulfur interaction with Cys 145 together with many Van der Waals interactions (Figure 4). Meanwhile, DNA-gyrase ligand (levofloxacin) forms H-bonds and salt bridges with Lys 415 and Arg 456 in addition to the formation of π-alkyl interaction with Arg 456 and C-H bond with Glu 474 and Glu 475. Regarding topoisomerase IV ligand (moxifloxacin), it forms three H-bonds, π-π bond, and alkyl-alkyl interaction with Arg 456, C-H bond, and many Van der Waals interactions with the amino acid residues at the active site. However, SARS-CoV-2 M^Pro^ ligand (FHR/PRD_002347; (∼{N}-[(2∼{S})-3-cyclohexyl-1-oxidanylidene-1-[[(2∼{S})-1-oxidanylidene-3-[(3∼{S})-2-oxidanylidenepyrrolidin-3-yl]propan-2-yl]amino]propan-2-yl]-1∼{H}-indole-2-carboxamide) revealed the formation of H-bond with Glu 166 and C-H bonds with His 164, His 41, Asn 142, π-alkyl interaction with Pro 168 and alkyl-alkyl interaction with Cys 145, and many Van der Waals interactions (Supplementary Figure S21).

Quercetin 7-O-*β*-D-glucoside revealed the highest fitting to dihydrofolate reductase owing to the formation of three H-bonds with Ile 94, Tyr 100, and Ala7 and four π-alkyl interactions with Leu 57, Val 54, Leu 50, and C-H bonds with Asp 27 and Trp 6 and the formation of two attractive charges with Arg 960 and Arg 32 (Figure 5). However, dihydrofolate reductase ligand (trimethoprim) forms three H-bonds with Ala 7, Ile 94, and π-alkyl interaction with Leu 50 and Ile 20 and C-H bonds with Asp 27, Trp 6, and Tyr 100 (Supplementary Figure S22).

Kaempferol showed an excellent fitting with the active site of *β*-lactamase due to the formation of H-bonds with Thr 253 and Thr 251, π-alkyl interaction with Ile 117, and C-H bonds with Gly 252 and Ser 84 together with the formation of π-cation and attractive charges with Lys 87 and Lys 250. In terms of SARS-CoV-2PL^pro^ and SARS-CoV-2 3CL^pro^, it forms a considerable number of bonds with the former represented by one H-bond with Asp 165, π-π bond with Tyr 269, amide–π bond with Gly 164, a salt bridge with Lys 158, π-lone pair bond with Leu 163, C-H bond with Gly 164, and π-donor H-bond with Leu 163. Meanwhile, it forms with SARS-CoV-2 3CL^pro^ three H-bonds with Glu 166, Asp 187, and Cys 145, π-π bond with His 41, π-alkyl bond with Met 49, π-sulfur bond with Cys 44 and Cys 145, and many Van der Waals interactions (Figure 6). *β*-Lactamase ligand (cefuroxime) forms five H-bonds with Lys 87, Ser 84, Ser 142, Thr 253, and Ile 117 in addition to three C-H bonds with Pro 183 and Gly 52 together with the formation of two attractive charges with Arg 236 and Lys 250. Regarding SARS-CoV-2PL^pro^ ligand S88 (N-[(3-fluorophenyl) methyl]-1-[(1R)-1-naphthalen-1-ylethyl]piperidine-4-carboxamid, it forms H-bonds with Tyr 269 and Lys 158, alkyl-alkyl interactions with Pro 249 and Tyr 265, π-alkyl interactions with Pro248 and Pro 249, π-π interactions with Tyr 269, C-H bonds with Gln 276, Tyr274, and Asp 165, in addition to the formation of the bond between Leu 163 and the halogen (Fluorine). SARS-CoV-2 3CL^pro^ ligand 3WL (5,6,7-trihydroxy-2-phenyl-4H-chromen-4-one) forms H-bonds with Leu 141, Ser144, Cys 145, and Gly 143, π-π bond with His 41, π-alkyl bond with Met 49 in addition to π-sulfur bond with Cys 145, C-H bonds with His 163 and Asn 142, and many Van der Waals interactions (Supplementary Figure S23). Interaction of all the metabolites with the different amino acid residues via the formation of various bonds with the active sites of proteins was illustrated in Supplementary Figures S24–S30.

## Conclusion

The antimicrobial and antiviral properties of the methanol leaf extract of *Buddleja indica* Lam (Scrophulariaceae) were studied. The plant showed potent antimicrobial antituberculous, anti-*Helicobacter pylori*, and antiviral activity owing to its richness with polyphenolic compounds represented mainly by phenolic acids and flavonoids. Molecular modeling showed that caffeic acid, quercetin 7-O-*β*-D-glucoside, and kaempferol showed the highest fitting score in proteins implicated in the incidence of bacterial infection and the occurrence and progression of SARS-CoV-2 that triggered the COVID-19 pandemic. Thus, it can be considered as a future source of cheap, substantially safe, and credible antibacterial, antifungal, and antiviral candidate of natural origin. Besides, these findings provide a scientific consolidation in support of the ethnomedicinal uses of many *Buddleja* species as a topical antiseptic. However, future clinical trials are warranted for further assuring the activity as an antibacterial agent and in solving the COVID-19 crises for the whole extract and its isolated compounds as well.

## Data Availability

The original contributions presented in the study are included in the article/Supplementary Material; further inquiries can be directed to the corresponding authors.
