# Supplementary material for: Phytoconstituents, In Vitro Anti-Infective Activity of Buddleja indica Lam., and In Silico Evaluation of its SARS-CoV-2 Inhibitory Potential
Source: Front Pharmacol. 2021 Apr 12;12:619373. doi: 10.3389/fphar.2021.619373 (PMC8072666; doi:10.3389/fphar.2021.619373)
Supplement: Supplementary file 1 [file datasheet1.docx]

**Supplementary data**

**Phytoconstituents, In Vitro Anti-Infective Activity of *Buddleja indica* Lam., and In Silico Evaluation of its SARS-CoV-2 Inhibitory Potential**

**Fadia S. Youssef, Ahmed E. Altyar, Abdelsattar M. Omar and Mohamed L. Ashour**

**Figure S1:** A scheme representing the isolation of compounds **(1-6)** from the *B .indica* total methanol leaves extract

**Figure S2-S19:** 1D and 2D spectral data for most of the isolated compounds

**Figure S20**. Validation of the docking experiments for DNA-gyrase (A), topoisomerase IV (B), dihydrofolate reductase (C), *β*-lactamase (D), SARS-CoV-2 M^Pro^ (E), SARS-CoV-2PL^pro^ (F) and SARS-CoV-2 3CL^pro^ (G)

**Figure S21.** 2D and 3D binding modes of (A) DNA-gyrase ligand (Levofloxacin), (B) Topoisomerase IV ligand (Moxifloxacin) and (C) SARS-CoV-2 M^Pro^ ligand (FHR/PRD_002347) in active sites of their respective enzymes

**Figure S22.** 2D and 3D binding modes of dihydrofolate reductase ligand (Trimethoprim) in dihydrofolate reductase active sites

**Figure S23**. 2D and 3D binding modes of (A) *β*-lactamase ligand (Cefuroxime), (B) SARS-CoV-2PL^pro^ ligand (S88) and (C) SARS-CoV-2 3CL^pro^ ligand (3WL) in active sites of their respective enzymes

**Figure S24**. 2D binding modes of *p*-hydroxy benzoic acid **(A),** quercetin 3-*O*- *β*-D-glucoside-7-*O*-α-L-rhamnoside **(B)** kaempferol 3-*O*- *β*-D glucoside-7-*O*- α-L-rhamnoside **(C),** quercetin 7-*O*- *β*-D glucoside **(D)** and kaempferol **(E)** in active sites of DNA-gyrase; dotted green lines indicate H-bonds; dotted light green lines indicate C-H-bonds; dotted purple lines indicate π-bonds; dotted orange bonds indicate salt bridge formation

**Figure S25**. 2D binding modes of *p*-hydroxy benzoic acid **(A),** quercetin 3-*O*- *β*-D-glucoside-7-*O*-α-L-rhamnoside **(B)** kaempferol 3-*O*- *β*-D glucoside-7-*O*- α-L-rhamnoside **(C),** quercetin 7-*O*- *β*-D glucoside **(D)** and kaempferol **(E)** in active sites of topoisomerase IV; dotted green lines indicate H-bonds; dotted light green lines indicate C-H-bonds; dotted purple lines indicate π-bonds; dotted orange bonds indicate salt bridge formation

**Figure S26**. 2D binding modes of *p*-hydroxy benzoic acid **(A),** quercetin 3-*O*- *β*-D-glucoside-7-*O*-α-L-rhamnoside **(B)** kaempferol 3-*O*- *β*-D glucoside-7-*O*- α-L-rhamnoside **(C),** quercetin 7-*O*- *β*-D glucoside **(D)** and caffeic acid l **(E)** in active sites of SARS-CoV-2 3CL^pro^; dotted green lines indicate H-bonds; dotted light green lines indicate C-H-bonds; dotted purple lines indicate π-bonds; dotted orange bonds indicate salt bridge formation

**Figure S27**. 2D binding modes of *p*-hydroxy benzoic acid **(A),** quercetin 3-*O*- *β*-D-glucoside-7-*O*-α-L-rhamnoside **(B)** kaempferol 3-*O*- *β*-D glucoside-7-*O*- α-L-rhamnoside **(C),** quercetin 7-*O*- *β*-D glucoside **(D)** and kaempferol **(E)** in active sites of SARS-CoV-2 M^Pro^; dotted green lines indicate H-bonds; dotted light green lines indicate C-H-bonds; dotted purple lines indicate π-bonds; dotted orange bonds indicate salt bridge formation

**Figure S28**. 2D binding modes of *p*-hydroxy benzoic acid **(A),** quercetin 3-*O*- *β*-D-glucoside-7-*O*-α-L-rhamnoside **(B)** kaempferol 3-*O*- *β*-D glucoside-7-*O*- α-L-rhamnoside **(C),** quercetin 7-*O*- *β*-D glucoside **(D)** and caffeic acid l **(E)** in active sites of *β*-lactamase; dotted green lines indicate H-bonds; dotted light green lines indicate C-H-bonds; dotted purple lines indicate π-bonds; dotted orange bonds indicate salt bridge formation

**Figure S29**. 2D binding modes of *p*-hydroxy benzoic acid **(A),** quercetin 3-*O*- *β*-D-glucoside-7-*O*-α-L-rhamnoside **(B)** kaempferol 3-*O*- *β*-D glucoside-7-*O*- α-L-rhamnoside **(C),** quercetin 7-*O*- *β*-D glucoside **(D)** and caffeic acid l **(E)** in active sites of SARS-CoV-2PL^pro^; dotted green lines indicate H-bonds; dotted light green lines indicate C-H-bonds; dotted purple lines indicate π-bonds; dotted orange bonds indicate salt bridge formation; red bonds; unfavorable interaction

**Figure S30**. 2D binding modes of *p*-hydroxy benzoic acid **(A),** quercetin 3-*O*- *β*-D-glucoside-7-*O*-α-L-rhamnoside **(B)** kaempferol 3-*O*- *β*-D glucoside-7-*O*- α-L-rhamnoside **(C)** kaempferol **(D)** and caffeic acid  **(E)** in active sites of dihydrofolate reductase; dotted green lines indicate H-bonds; dotted light green lines indicate C-H-bonds; dotted purple lines indicate π-bonds; dotted orange bonds indicate salt bridge formation; red bonds; unfavorable interaction

**Table S1** Effect of different BIT concentrations on the viral replication using the direct plaque reduction assay

**Table S2** Inhibitory percentage of BIT and isoniazid (standard) against *Mycobacterium tuberculosis* growth

**Table S3** Inhibitory percentage of BIT and Clarithromycin (standard) against *Helicobacter pylori growth*

**
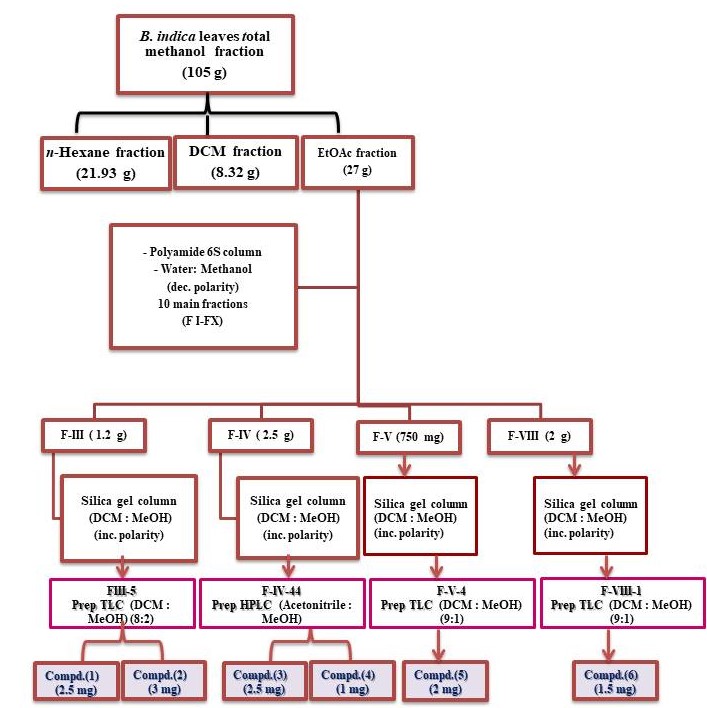
**

**Figure S1:** A scheme representing the isolation of compounds **(1-6)** from the *B .indica* Lam. total methanol leaves extract

Fig. S2: ^1^H NMR spectrum for *p*-hydroxy benzoic acid **(1)**

Fig. S3: APT NMR spectrum for *p*-hydroxy benzoic acid **(1)**

Fig. S4: ^1^H NMR spectrum for caffeic acid **(2)**

Fig. S5: APT NMR spectrum for caffeic acid **(2)**

Fig. S6: ^1^H-^1^H COSY spectrum for caffeic acid **(2)**

Fig. S7: HSQC spectrum for caffeic acid **(2)**

Fig. S8: HMBC spectrum for caffeic acid **(2)**

Fig. S9: ^1^H NMR spectrum for quercetin 3-*O*- *β-D*-glucoside-7-*O*-*α*-L-rhamnoside **(3)**

Fig. S10: APT- NMR spectrum for quercetin 3-*O*- *β-D*-glucoside-7-*O*-*α*-L-rhamnoside **(3)**

Fig. S11: ^1^H NMR spectrum for kaempferol 3-*O*- *β-D*-glucoside-7-*O*-*α*-L-rhamnoside **(4)**

Fig. S12: APT^-^-NMR spectrum for kaempferol 3-*O*- *β-D*-glucoside-7-*O*-*α*-L-rhamnoside **(4)**

Fig. S13: ^1^H-^1^H COSY spectrum for kaempferol 3-*O*- *β-D*-glucoside-7-*O*-*α*-L-rhamnoside **(4)**

Fig. S14: HSQC spectrum for kaempferol 3-*O*- *β-D*-glucoside-7-*O*-*α*-L-rhamnoside **(4)**

Fig. S15: HMBC spectrum for kaempferol 3-*O*- *β-D*-glucoside-7-*O*-*α*-L-rhamnoside **(4)**

Fig. S16: ^1^H NMR spectrum for quercetin 7-*O*- *β*-D-glucoside **(5)**

Fig. S17: APT- NMR spectrum for quercetin 7-*O*- *β*-D-glucoside **(5)**

Fig. S18: ^1^H NMR spectrum for kaempferol **(6)**

Fig. S19: ^1^H NMR spectrum for kaempferol **(6)**

**Figure S20**. Validation of the docking experiments for DNA-gyrase (A), topoisomerase IV (B), dihydrofolate reductase (C), *β*-lactamase (D), SARS-CoV-2 M^Pro^ (E), SARS-CoV-2PL^pro^ (F) and SARS-CoV-2 3CL^pro^ (G)


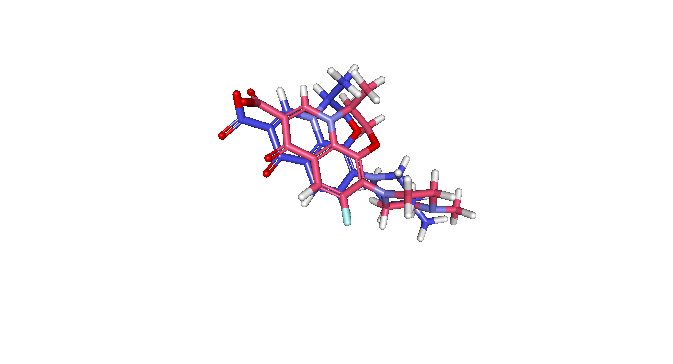

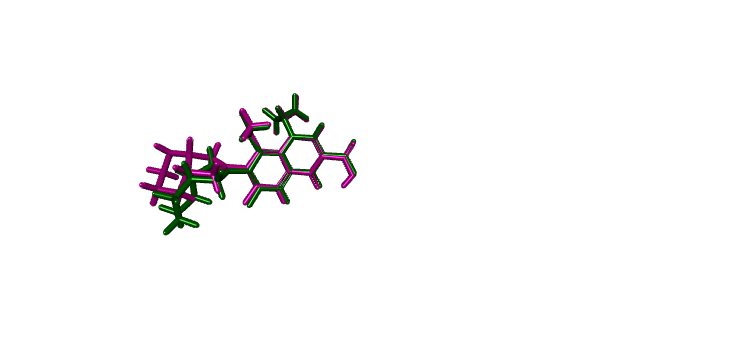

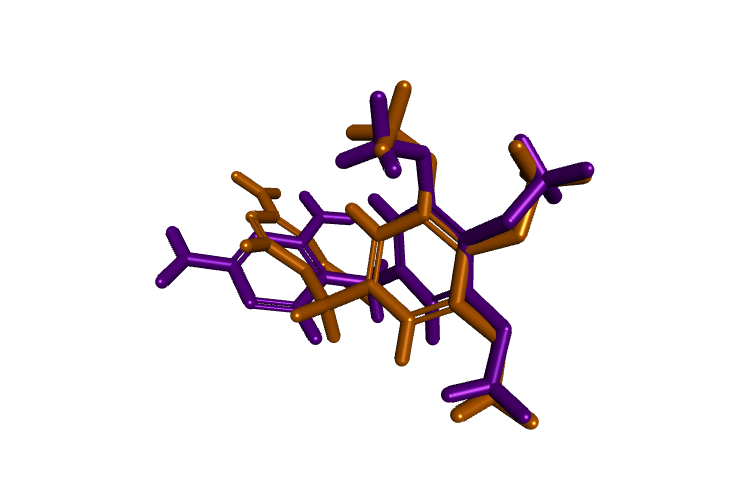

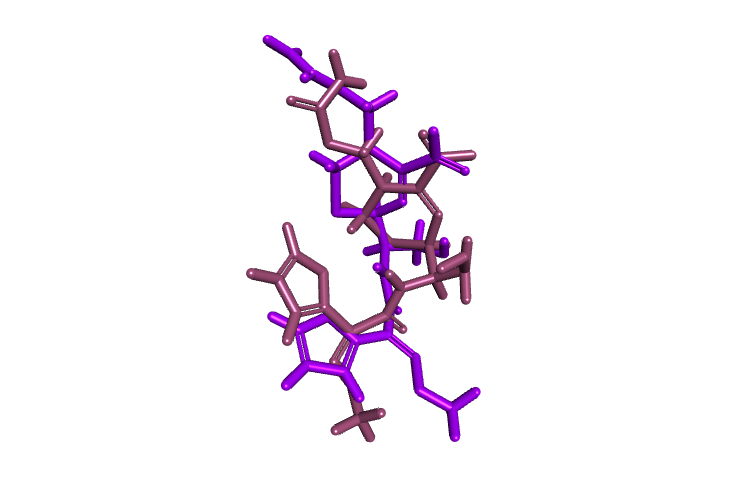

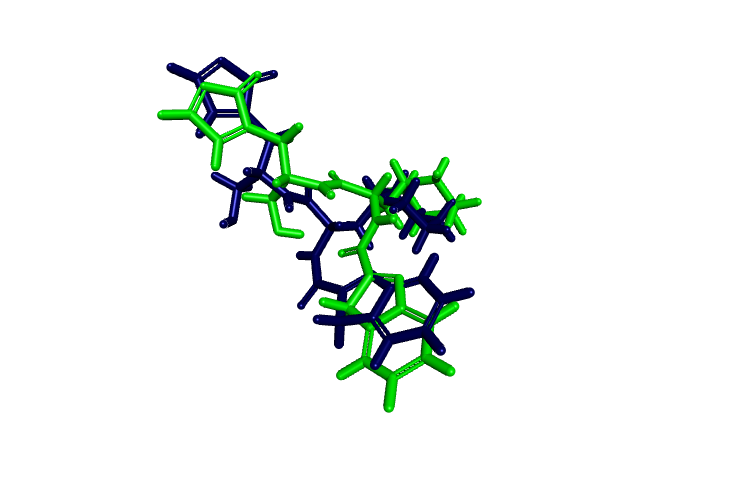

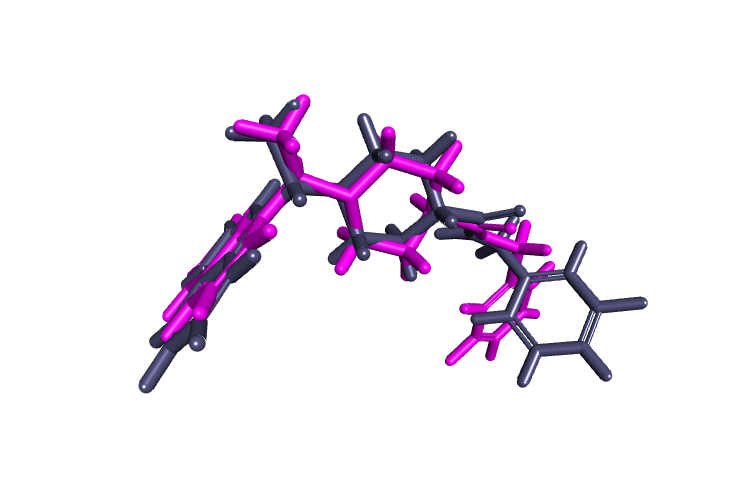

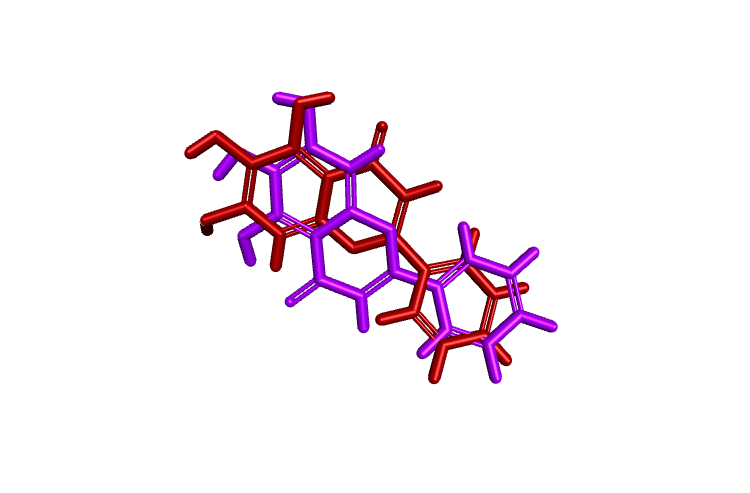


**(A)**

**(B)**

**(C)**

**(D)**

**(E)**

**(F)**

**(G)**

**(G)**


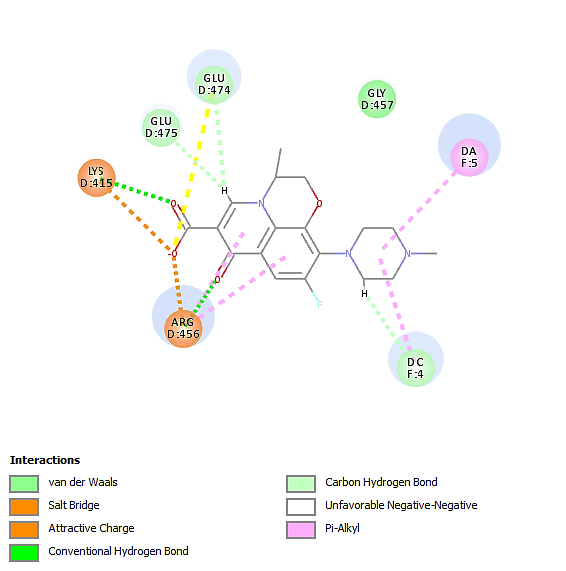

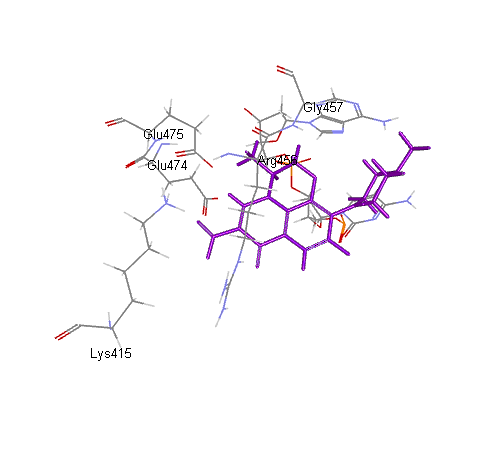

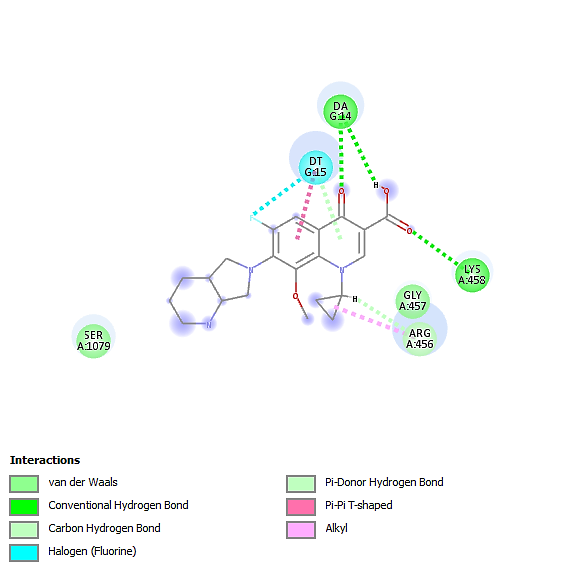

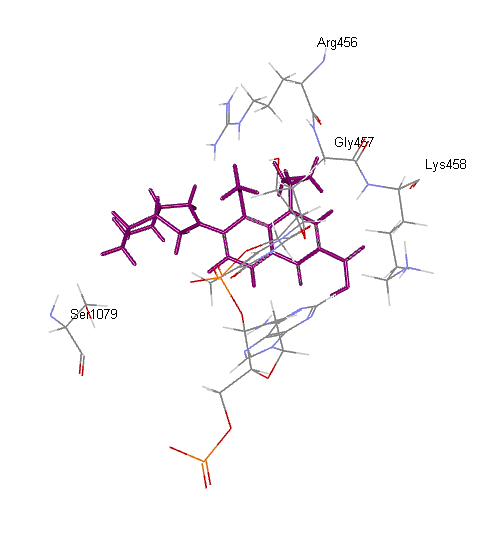

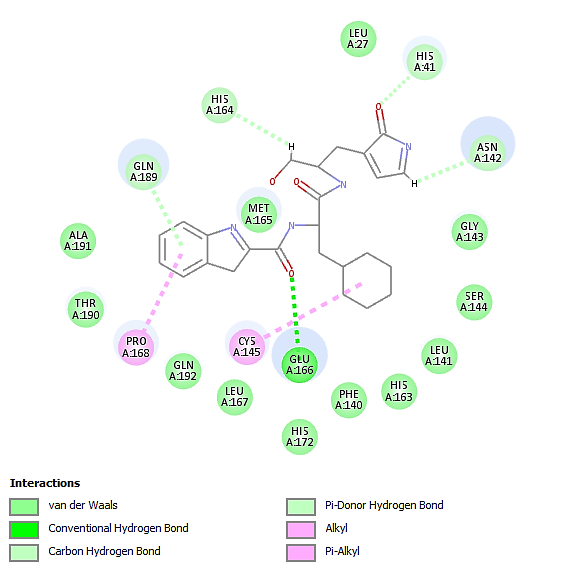

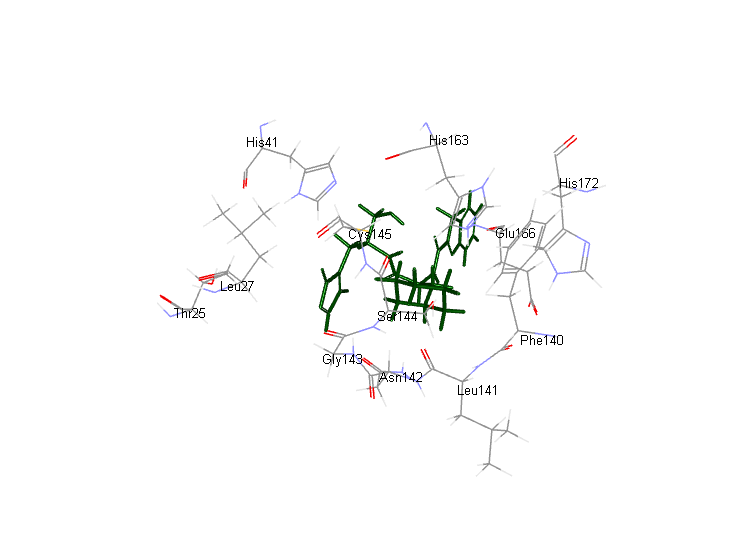


**(A)**

**(B)**

**(C)**

**Figure S21.** 2D and 3D binding modes of (A) DNA-gyrase ligand (Levofloxacin), (B) Topoisomerase IV ligand (Moxifloxacin) and (C) SARS-CoV-2 M^Pro^ ligand (FHR/PRD_002347) in active sites of their respective enzymes


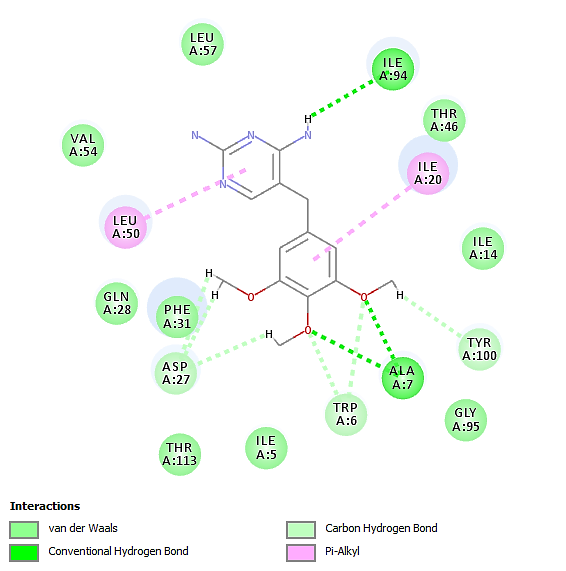

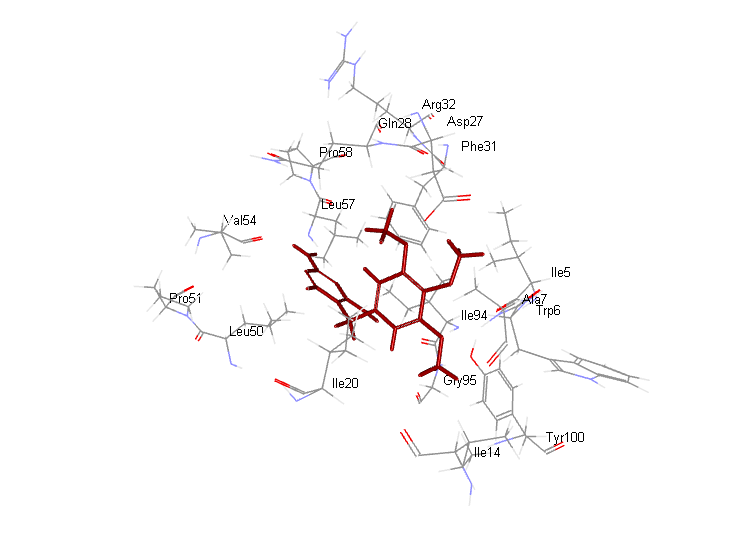


**Figure S22.** 2D and 3D binding modes of dihydrofolate reductase ligand (Trimethoprim) in dihydrofolate reductase active sites


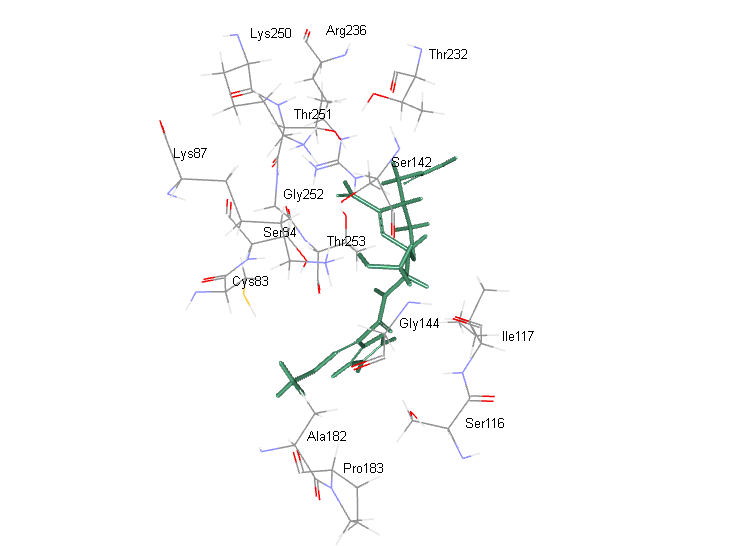

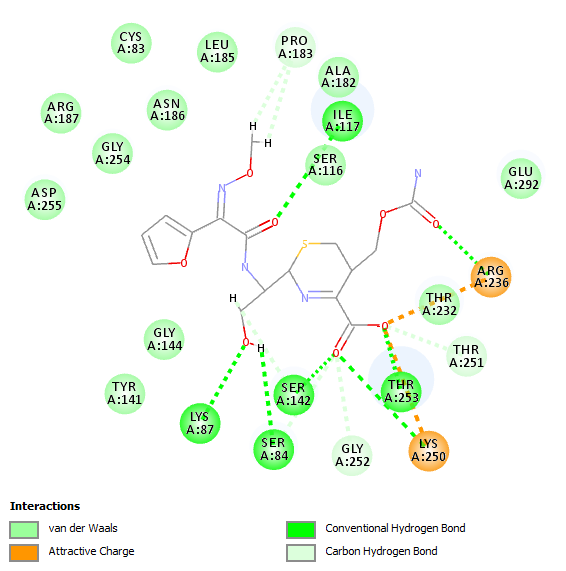

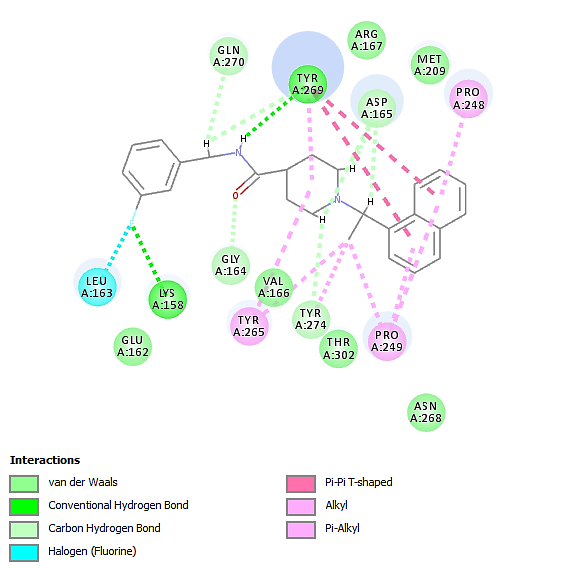

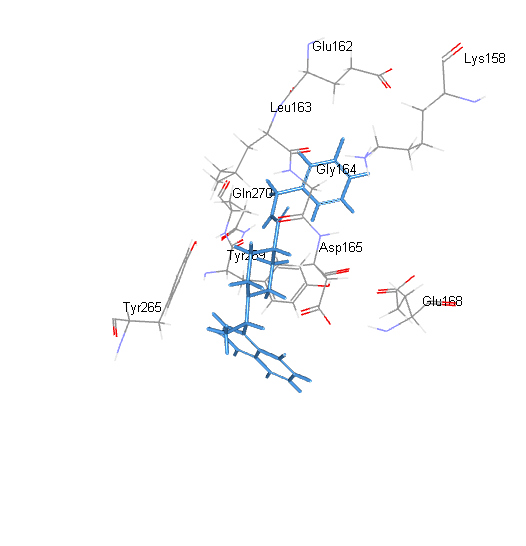

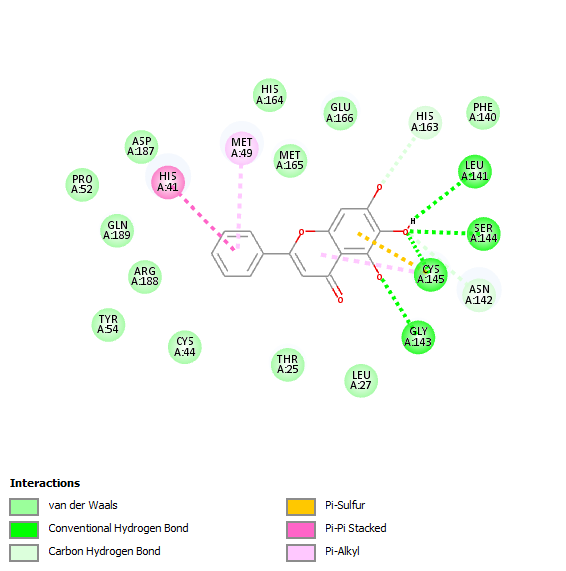

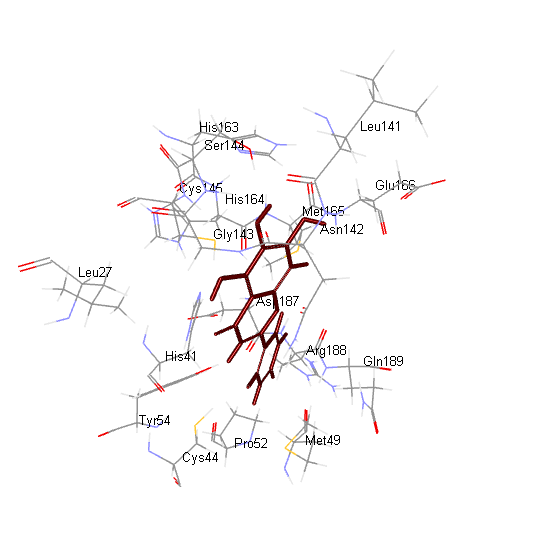


**(A)**

**(B)**

**(C)**

**Figure S23** . 2D and 3D binding modes of (A) *β*-lactamase ligand (Cefuroxime), (B) SARS-CoV-2PL^pro^ ligand (S88) and (C) SARS-CoV-2 3CL^pro^ ligand (3WL) in active sites of their respective enzymes


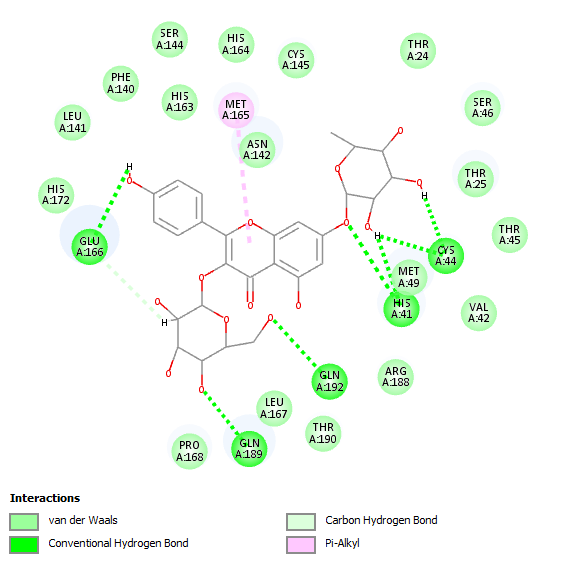

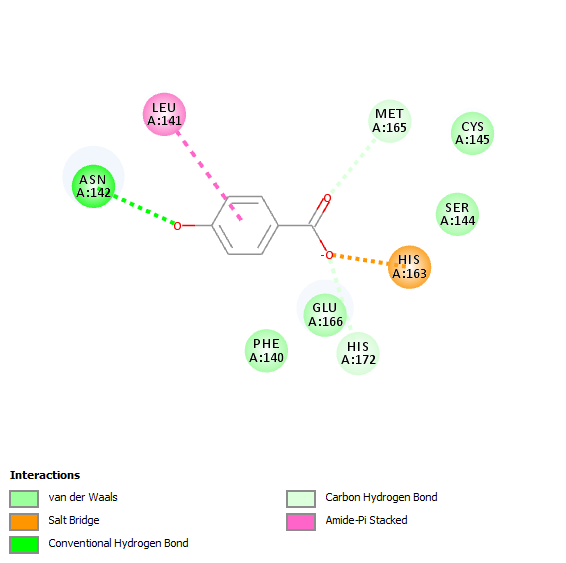


**(B)**

**(A)**


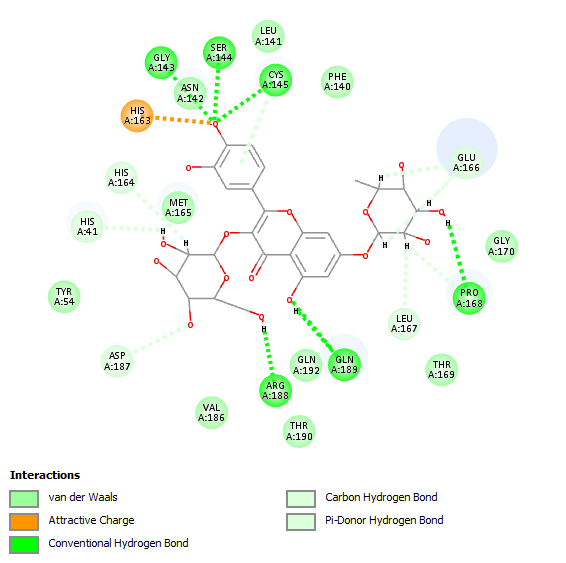


**(C)**


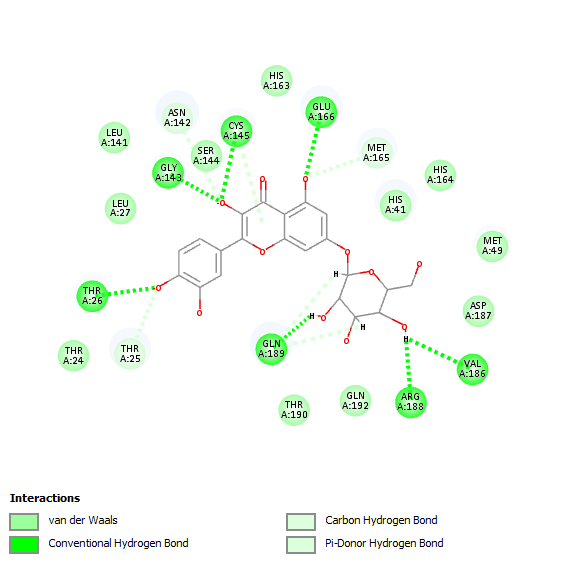


**(D)**


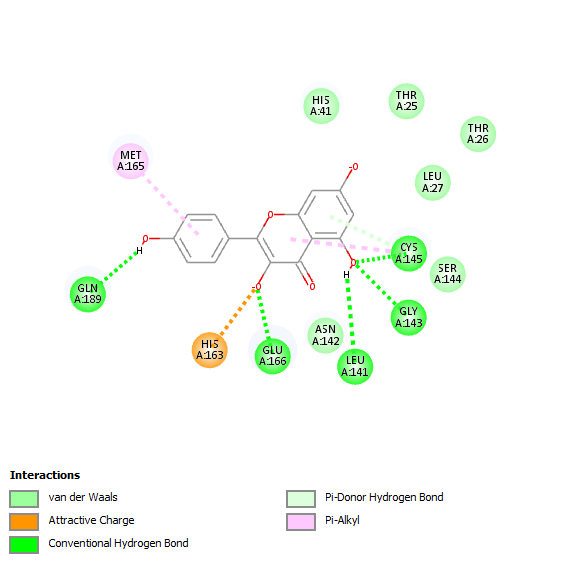


**(E)**

**Figure S24** . 2D binding modes of *p*-hydroxy benzoic acid **(A),** quercetin 3-*O*- *β*-D-glucoside-7-*O*-α-L-rhamnoside **(B)** kaempferol 3-*O*- *β*-D glucoside-7-*O*- α-L-rhamnoside **(C),** quercetin 7-*O*- *β*-D glucoside **(D)** and kaempferol **(E)** in active sites of DNA-gyrase; dotted green lines indicate H-bonds; dotted light green lines indicate C-H-bonds; dotted purple lines indicate π-bonds; dotted orange bonds indicate salt bridge formation

**
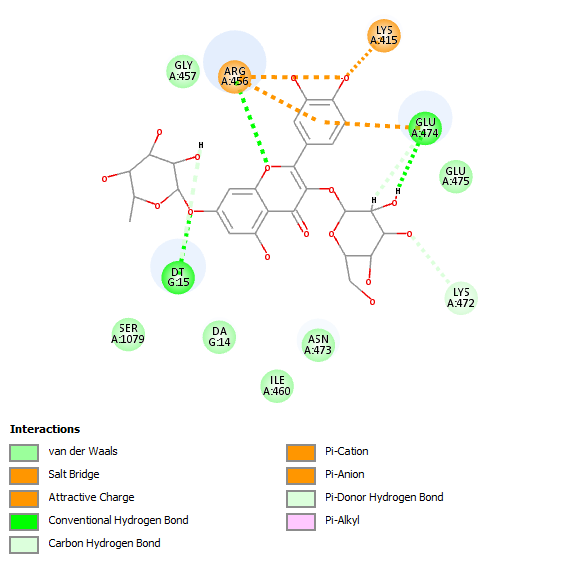
**
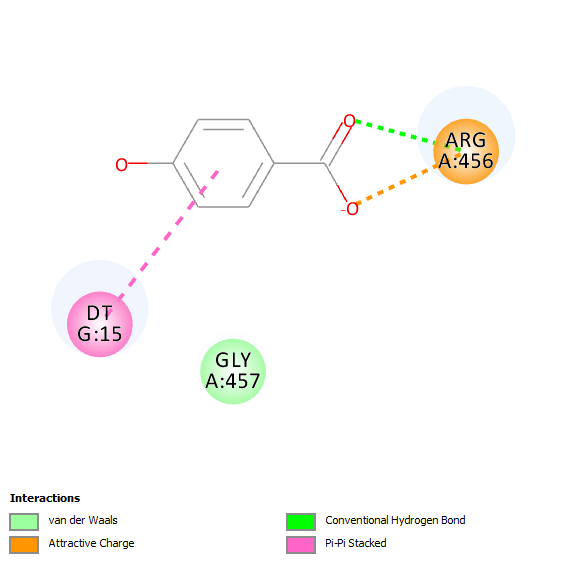


**(C)**

**(B)**

**(A)**

**
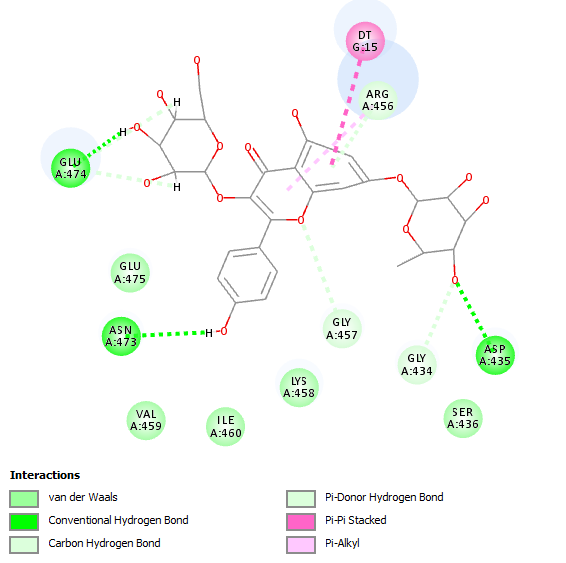
**

**
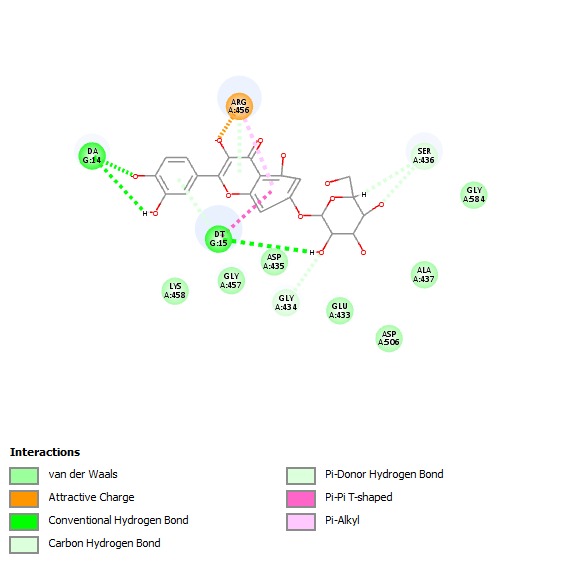
**

**(D)**

**
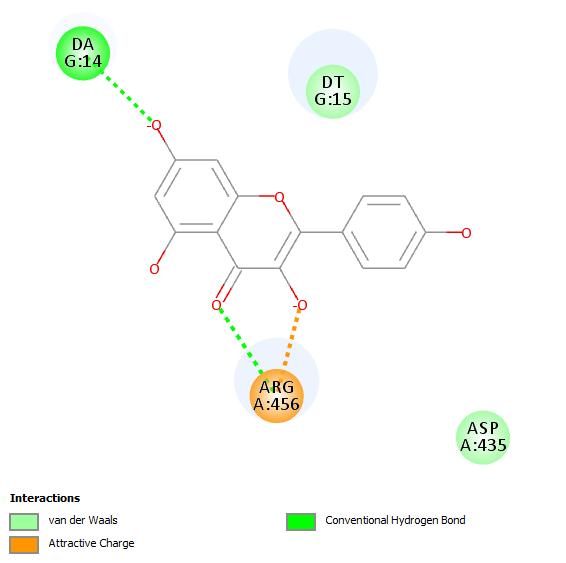
**

**(E)**

**Figure S25** . 2D binding modes of *p*-hydroxy benzoic acid **(A),** quercetin 3-*O*- *β*-D-glucoside-7-*O*-α-L-rhamnoside **(B)** kaempferol 3-*O*- *β*-D glucoside-7-*O*- α-L-rhamnoside **(C),** quercetin 7-*O*- *β*-D glucoside **(D)** and kaempferol **(E)** in active sites of topoisomerase IV; dotted green lines indicate H-bonds; dotted light green lines indicate C-H-bonds; dotted purple lines indicate π-bonds; dotted orange bonds indicate salt bridge formation


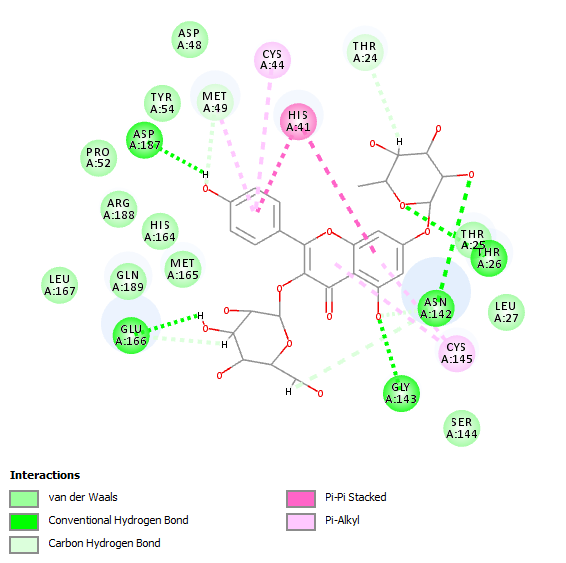

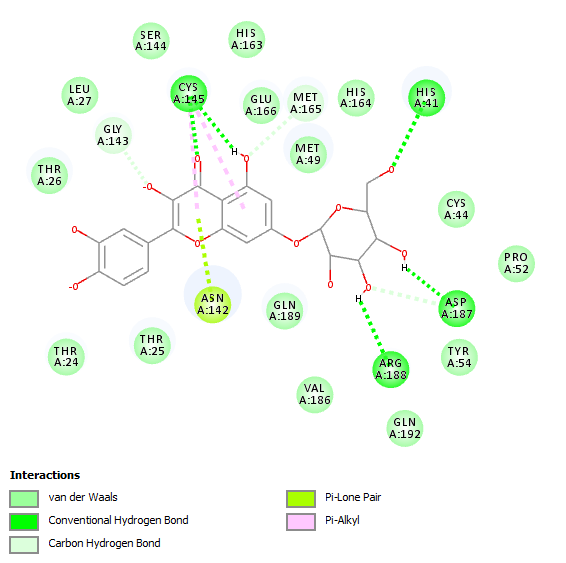

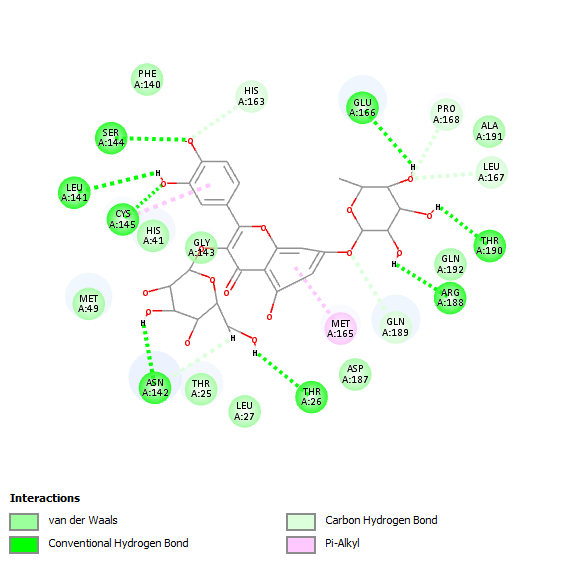
**
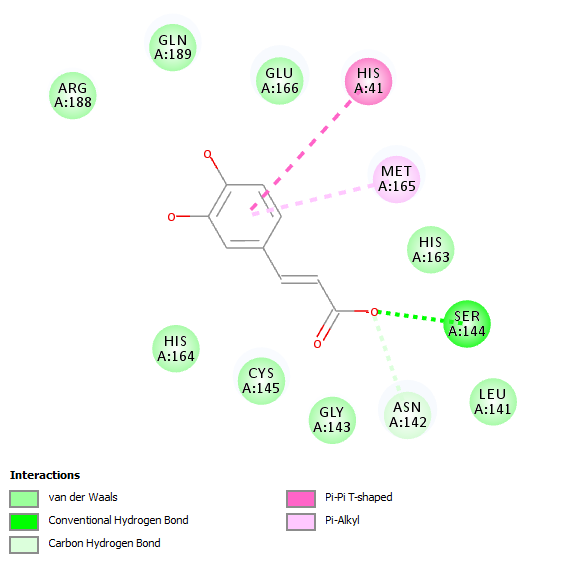
**

**(A)**

**(D)**

**(C)**

**(B)**


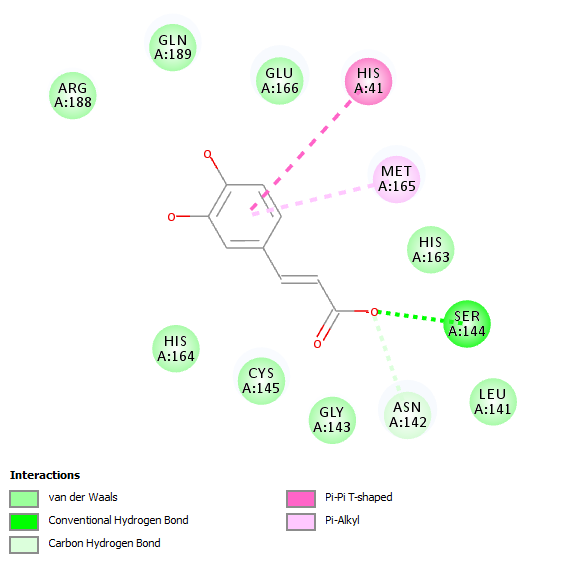


**(E)**

**Figure S26**. 2D binding modes of *p*-hydroxy benzoic acid **(A),** quercetin 3-*O*- *β*-D-glucoside-7-*O*-α-L-rhamnoside **(B)** kaempferol 3-*O*- *β*-D glucoside-7-*O*- α-L-rhamnoside **(C),** quercetin 7-*O*- *β*-D glucoside **(D)** and caffeic acid l **(E)** in active sites of SARS-CoV-2 3CL^pro^; dotted green lines indicate H-bonds; dotted light green lines indicate C-H-bonds; dotted purple lines indicate π-bonds; dotted orange bonds indicate salt bridge formation


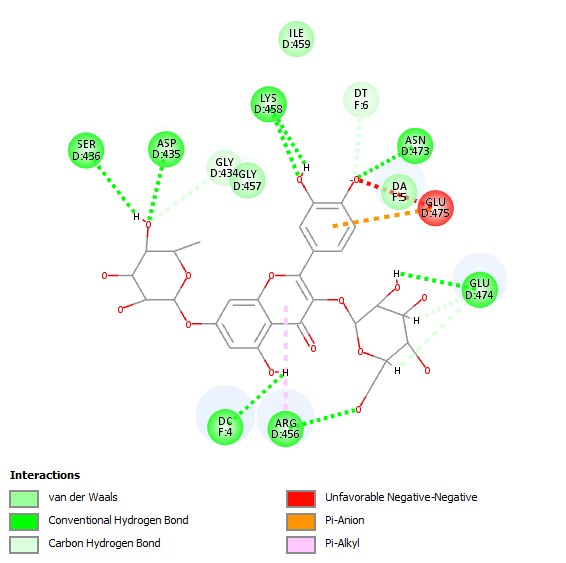

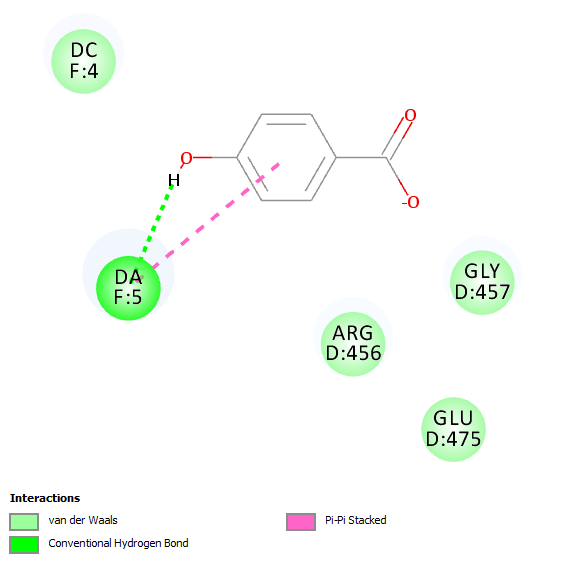


**(B)**

**(A)**


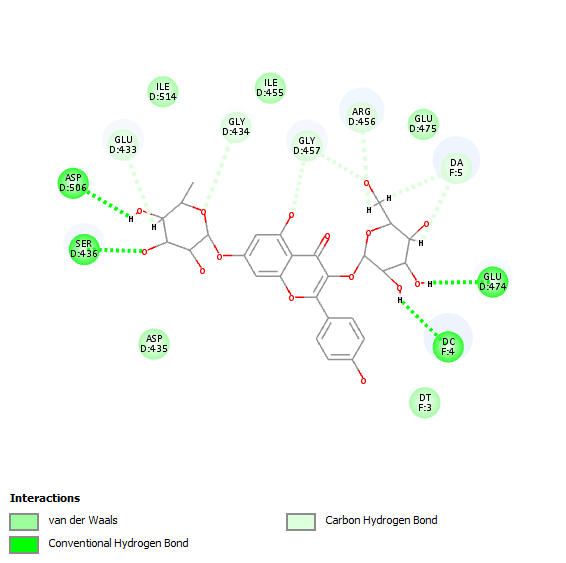


**(C)**

**(D)**


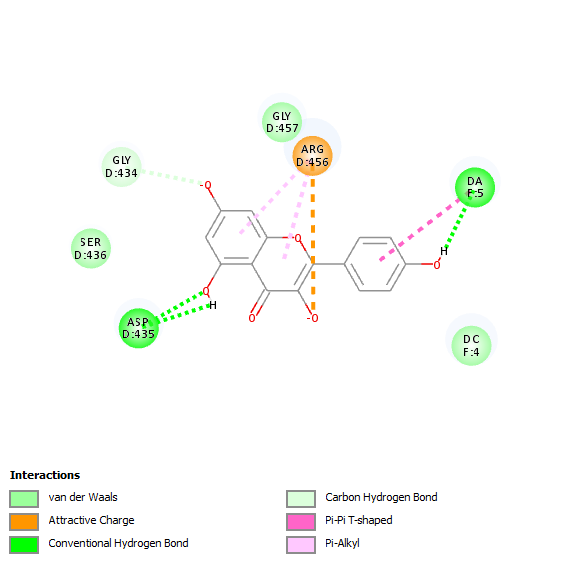

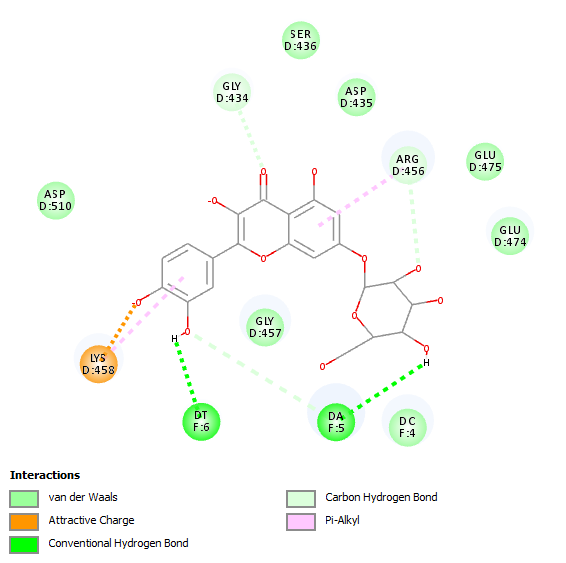


**(E)**

**Figure S27** 2D binding modes of *p*-hydroxy benzoic acid **(A),** quercetin 3-*O*- *β*-D-glucoside-7-*O*-α-L-rhamnoside **(B)** kaempferol 3-*O*- *β*-D glucoside-7-*O*- α-L-rhamnoside **(C),** quercetin 7-*O*- *β*-D glucoside **(D)** and kaempferol **(E)** in active sites of SARS-CoV-2 M^Pro^; dotted green lines indicate H-bonds; dotted light green lines indicate C-H-bonds; dotted purple lines indicate π-bonds; dotted orange bonds indicate salt bridge formation; red bonds; unfavorable interaction


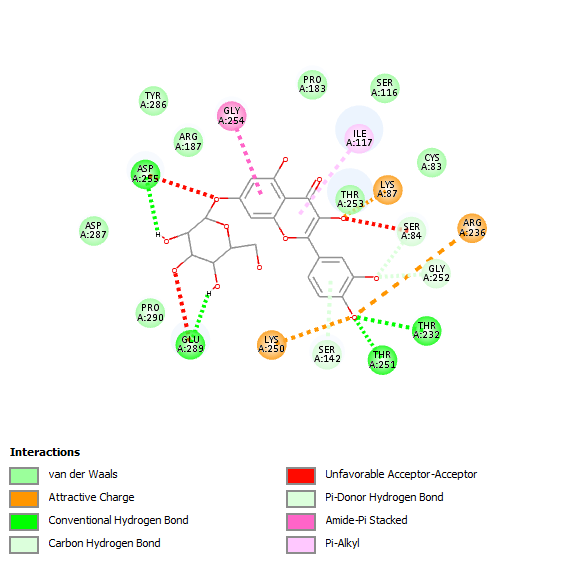

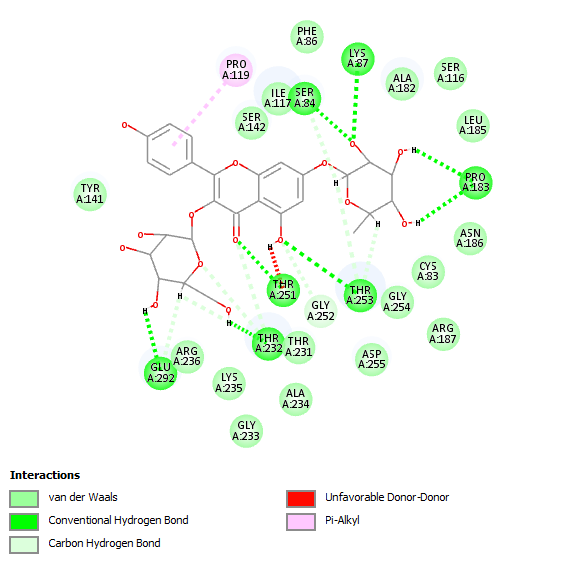

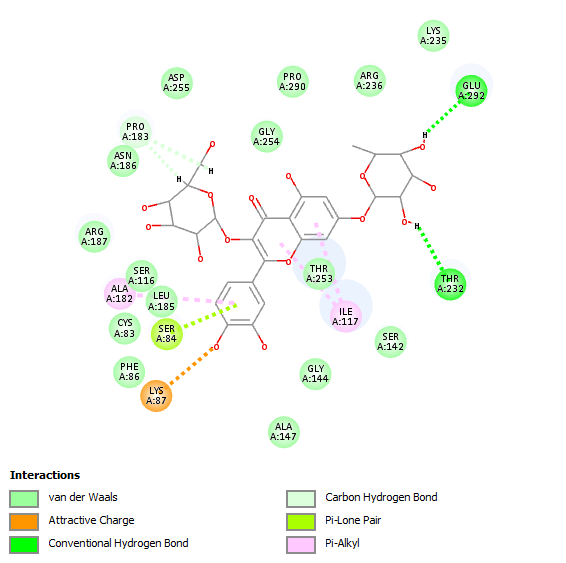

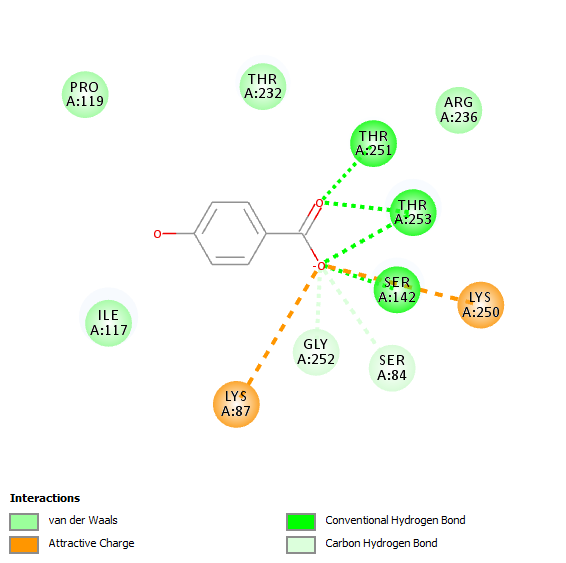


**(D)**

**(C)**

**(A)**

**(B)**


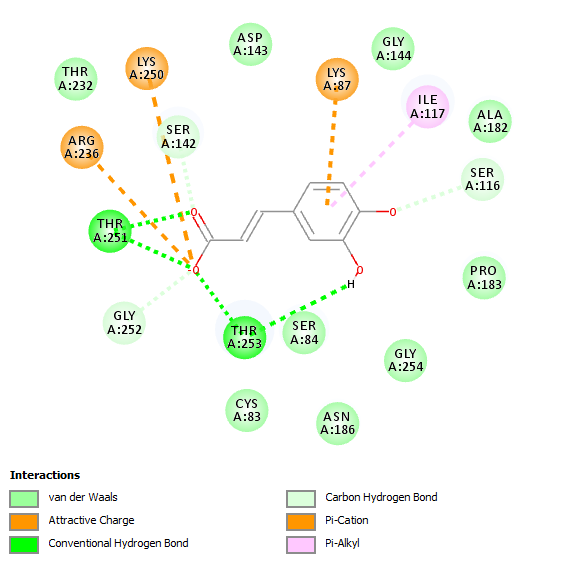


**(E)**

**Figure S28**. 2D binding modes of *p*-hydroxy benzoic acid **(A),** quercetin 3-*O*- *β*-D-glucoside-7-*O*-α-L-rhamnoside **(B)** kaempferol 3-*O*- *β*-D glucoside-7-*O*- α-L-rhamnoside **(C),** quercetin 7-*O*- *β*-D glucoside **(D)** and caffeic acid l **(E)** in active sites of *β*-lactamase; dotted green lines indicate H-bonds; dotted light green lines indicate C-H-bonds; dotted purple lines indicate π-bonds; dotted orange bonds indicate salt bridge formation; red bonds; unfavorable interaction


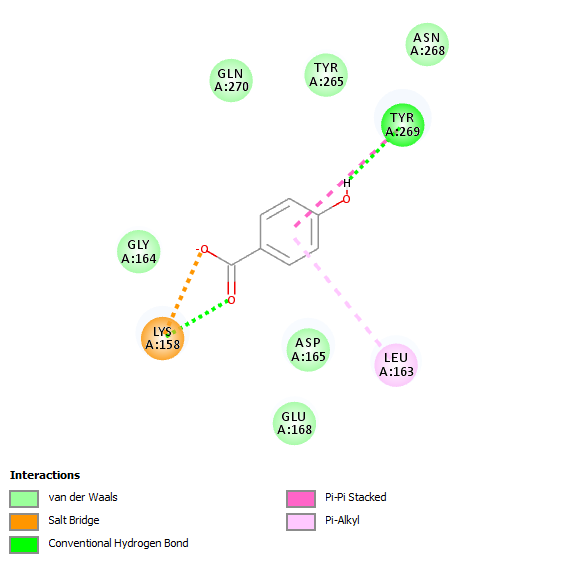

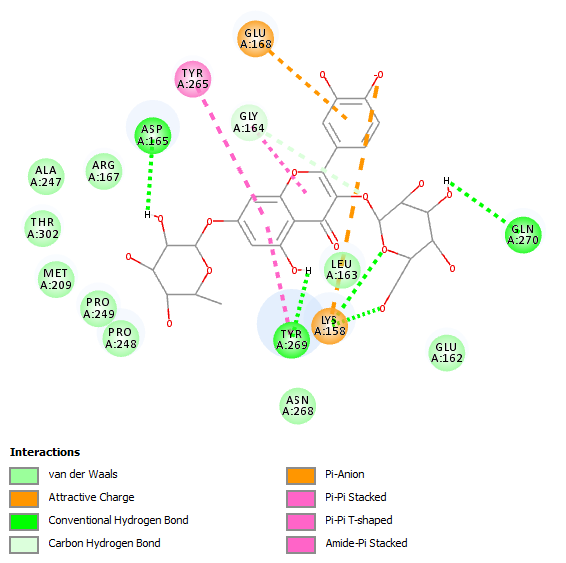


**(B)**

**(A)**


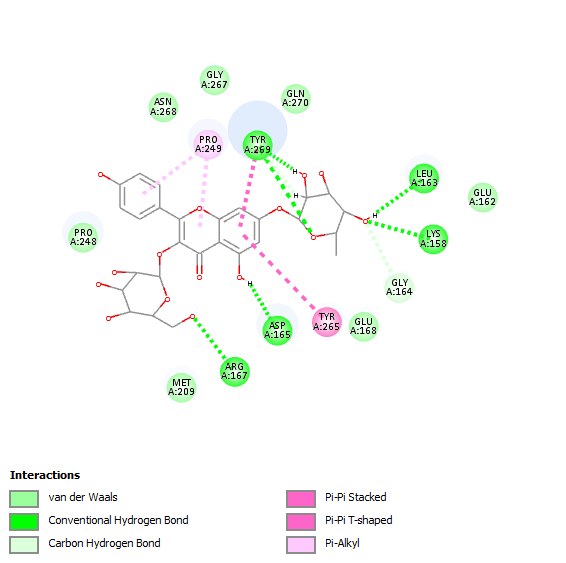
**
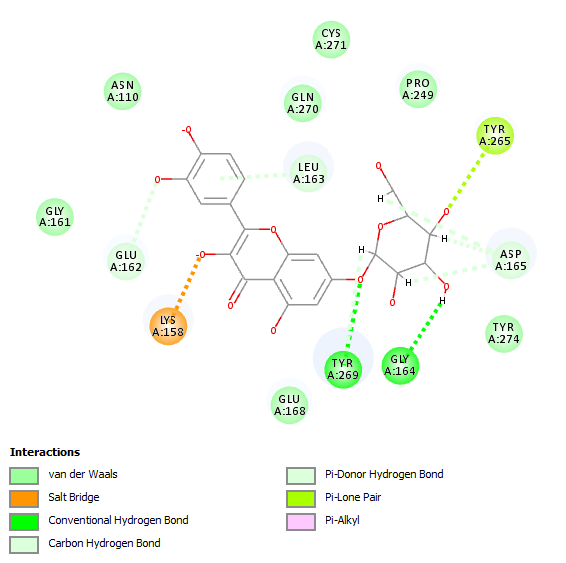
**

**(D)**

**(C)**


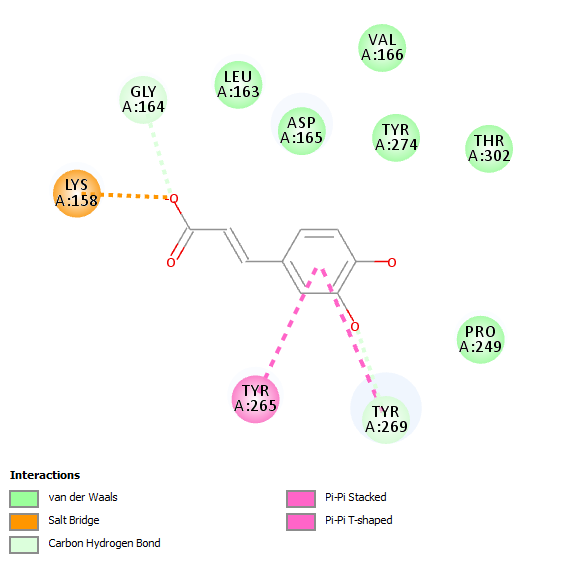


**(E)**

**Figure S29**. 2D binding modes of *p*-hydroxy benzoic acid **(A),** quercetin 3-*O*- *β*-D-glucoside-7-*O*-α-L-rhamnoside **(B)** kaempferol 3-*O*- *β*-D glucoside-7-*O*- α-L-rhamnoside **(C),** quercetin 7-*O*- *β*-D glucoside **(D)** and caffeic acid  **(E)** in active sites of SARS-CoV-2PL^pro^; dotted green lines indicate H-bonds; dotted light green lines indicate C-H-bonds; dotted purple lines indicate π-bonds; dotted orange bonds indicate salt bridge formation; red bonds; unfavorable interaction


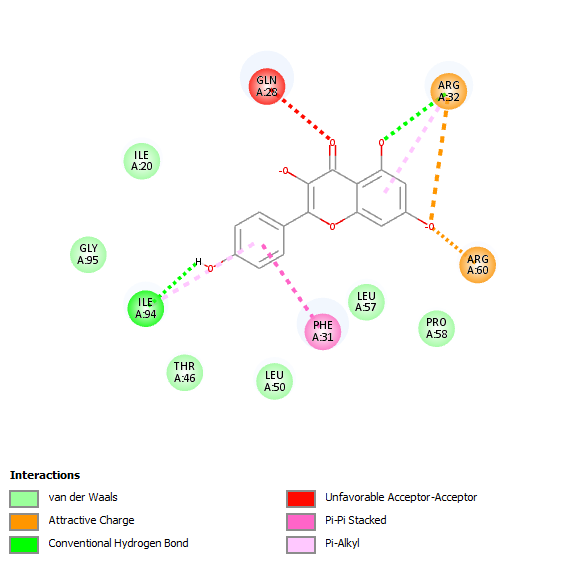

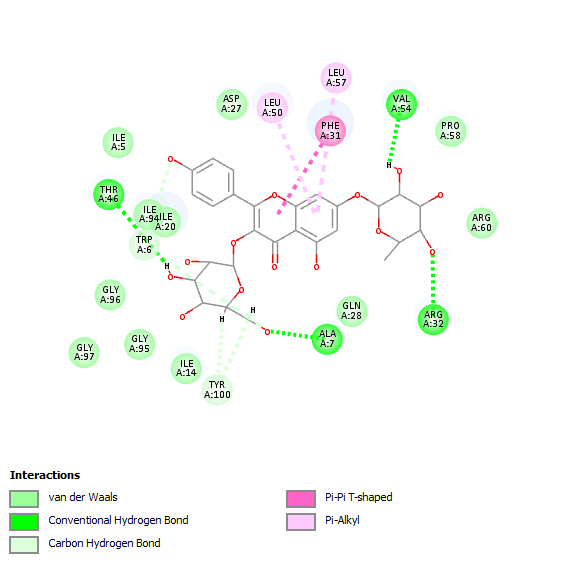

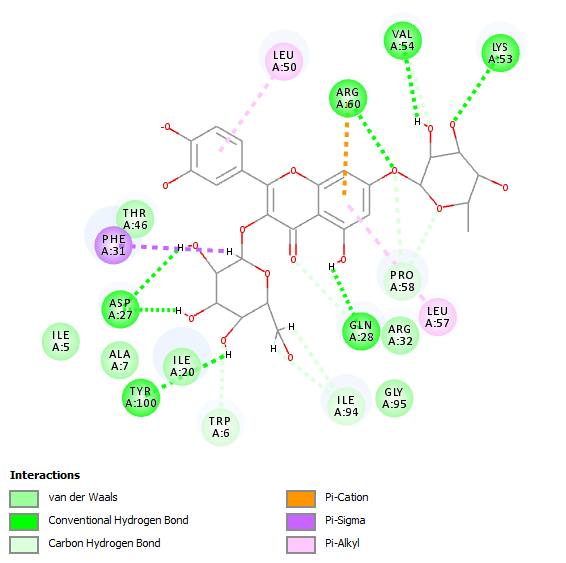

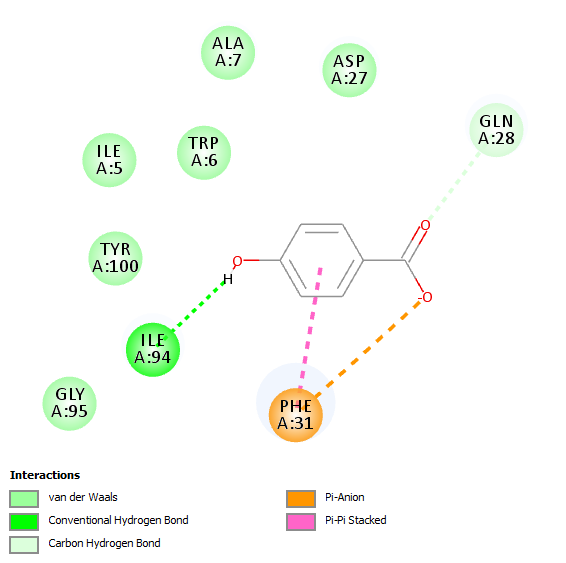


**(D)**

**(C)**

**(B)**

**(A)**


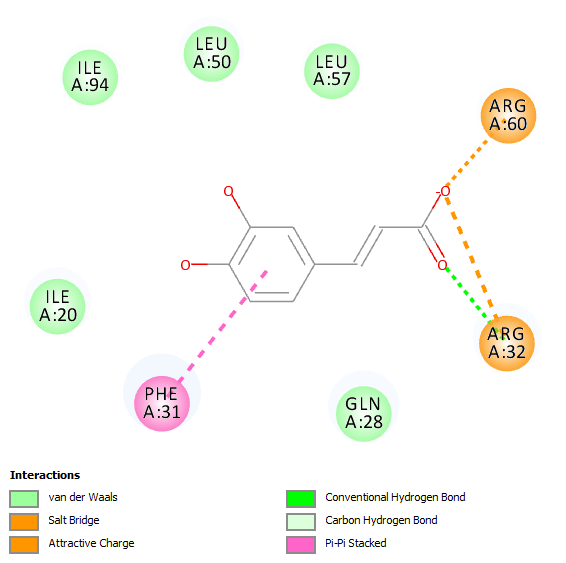


**Figure S30**. 2D binding modes of *p*-hydroxy benzoic acid **(A),** quercetin 3-*O*- *β*-D-glucoside-7-*O*-α-L-rhamnoside **(B)** kaempferol 3-*O*- *β*-D glucoside-7-*O*- α-L-rhamnoside **(C)** kaempferol **(D)** and caffeic acid  **(E)** in active sites of dihydrofolate reductase; dotted green lines indicate H-bonds; dotted light green lines indicate C-H-bonds; dotted purple lines indicate π-bonds; dotted orange bonds indicate salt bridge formation; red bonds; unfavorable interaction

**(E)**

**Table S1 Effect of different BIT concentration on the viral replication using the direct plaque reduction assay**

|  | **% Inhibition in viral replication** | |
| --- | --- | --- |
|  | HSV-1 | VSV |
| **BI extract** |  | |
| 6.25 μg/ml | 07.05 ± 1.16 | 12.18 ± 2.21 |
| 12.50 μg/ml | 11.63 ± 2.86 | 18.52 ± 1.70 |
| 25.00 μg/ml | 26.97 ± 3.09 | 32.54 ± 2.81 |
| 50.00 μg/ml | 41.85 ± 2.32 | 48.38 ± 2.51 |
| 100 μg/ml | 100 | 100 |

Data are presented as means ± S.D. n=3.

HSV-1: Herpes Simplex type-1 virus, VSV: Vesicular stomatitis virus

**Table S2 Inhibitory percentage of BIT and isoniazid (standard) against *Mycobacterium tuberculosis* growth**

| **Sample** | **BIT** | **Isoniazid** |
| --- | --- | --- |
| 200 µg/mL | 100± 0 | 100± 0 |
| 100 µg/mL | 100± 0 | 100± 0 |
| 50 µg/mL | 90.74± 1.2 | 100± 0 |
| 25 µg/mL | 79.4± 1.5 | 100± 0 |
| 12.5 µg/mL | 75.8± 0.72 | 93.24± 2.1 |
| **Minimum inhibitory concentration (µg/mL)** | **100** | **25** |

**Table S3 Inhibitory percentage of BIT and Clarithromycin (standard) against *Helicobacter pylori growth***

| **Sample** | **BIT** | **Isoniazid** |
| --- | --- | --- |
| 160 µg/mL | 100± 0 | 100± 0 |
| 80 µg/mL | 100± 0 | 100± 0 |
| 40 µg/mL | 94.58± 0.72 | 100± 0 |
| 20 µg/mL | 89.71± 1.2 | 100± 0 |
| 10 µg/mL | 73.4± 1.5 | 95.37± 2.1 |
| **Minimum inhibitory concentration (µg/mL)** | 80 | 20 |
